# A rapid transition from subduction to Barrovian metamorphism: geochronology of mafic–ultramafic relicts of oceanic crust in the Central Alps, Switzerland

**DOI:** 10.1186/s00015-024-00462-7

**Published:** 2024-08-30

**Authors:** Kim Lemke, Daniela Rubatto, Jörg Hermann

**Affiliations:** 1 Institute of Earth Sciences, University of Lausanne, Géopolis Building, 1015 Lausanne, Switzerland; 2 Institute of Geological Sciences, University of Bern, Baltzerstrasse 1+3, 3012 Bern, Switzerland

**Keywords:** Ophiolitic relicts, Oxygen isotopes, High-pressure melting, Adula nappe and Cima-Lunga unit, Zircon and rutile geochronology

## Abstract

Relicts of subducted oceanic lithosphere provide key information for the tectonic reconstructions of convergent margins. In the Central Alps, such relicts occur as isolated mafic–ultramafic lenses within the migmatites of the southern Adula nappe and Cima-Lunga unit. Analysis of the major-, minor-, and accessory minerals of these ophiolitic relicts, combined with zircon and rutile U–Pb ages and zircon oxygen isotopes, allows the reconstruction of different stages of their complex evolution. The mafic–ultramafic suite in Valle di Moleno consists of chlorite-harzburgites associated with metarodingites and retrogressed eclogites. Relic omphacite and kyanite in retrogressed eclogites provide evidence for subduction-related metamorphism. Increasing X_Prp_ in the garnet mantle towards the rim documents heating during high-pressure metamorphism up to 800–850 °C. Polyphase inclusions and chemical zoning in garnet suggest fluid-assisted melting during high-pressure metamorphism dated at 31.0 ± 0.9 Ma. In Val Cama, chlorite-harzburgites, metarodingites and calcsilicate-metasediments occur. Detrital zircon ages in the metasediment suggest a Mesozoic deposition. The metarodingite-metaperidotite-metasediment association and the low δ^18^O signatures of zircon (δ^18^O 3.0–3.7‰), inherited from seafloor metasomatism of the protoliths, show that the rocks are derived from former altered oceanic crust. Amphibolite facies metamorphism related to the Central Alps Barrovian evolution in Val Cama occurred at 28.8 ± 1.5 Ma. The combined data from Moleno and Cama indicate a rapid transition (~ 2 Ma) from subduction to collisional metamorphism with corresponding exhumation rates of 3–6 cm/year. Fast exhumation tectonics may have been favored by slab break-off or slab extraction. U–Pb dating of rutile from both localities yields ages of ~ 20 Ma, suggesting that these rocks remained at amphibolite-facies conditions for about 10 Ma and underwent a second fast exhumation of 3 cm/year associated with vertical movements along the Insubric line.

**Supplementary Information:**

The online version contains supplementary material available at 10.1186/s00015-024-00462-7.

## Introduction

The transition from subduction to collision tectonics is a crucial phase for the formation of mountain belts such as the Himalayas and the Alps. This stage occurs when the oceanic crust separating the continental blocks is removed and the continental margin enters the subduction zone. In the case of the Alps, this transition is reached when the distal European margin enters the subduction channel. The Adula nappe and Cima-Lunga unit in the Central Alps represent the outer European continental margin of the Piemont-Ligurian Ocean, which was subducted and exhumed during the Alpine orogeny (Schmid et al., 1996). In these units, remnants of the Piemont-Ligurian Ocean occur as isolated lenses or bodies, consisting of dehydrated serpentinites, such as Chl- or Grt- metaperidotites, metarodingites and partially retrogressed eclogites (e.g. Dale & Holland, 2003; Evans et al., 1979; Evans & Trommsdorff, 1978; Möckel, 1969; Pfiffner & Trommsdorff, 1998). The association of mafic–ultramafic lenses that are embedded in continental basement rocks consisting of paragneisses and orthogneisses has been interpreted as a former subduction channel (Engi et al., 2001). Some of the mafic–ultramafic lenses record subduction-related Alpine metamorphism at eclogite-facies conditions (Evans & Trommsdorff, 1978; Pfiffner & Trommsdorff, 1998) overprinted at amphibolite facies during the Barrovian stage of the Lepontine dome (Engi et al., 1995; Todd & Engi, 1997). Reconstructing the history of ophiolitic relicts in such small lenses is often challenging compared to coherent ophiolitic sequences such as the Saas-Zermatt Unit in the Western Alps (e.g. Amato et al., 1999; Angiboust et al., 2012; Barnicoat & Fry, 1986; Bucher et al., 2005; Rubatto et al., 1998). As the mafic–ultramafic rocks are associated with basement rocks, it is necessary to demonstrate that the metamorphism is of Alpine rather than pre-Alpine age. For example, eclogites of both Alpine and pre-Alpine age occur within the Adula nappe (Becker, 1993; Brouwer et al., 2005; Gebauer et al., 1996; Liati et al., 2009, Herwartz et al., 2011), and dating of eclogites is therefore essential to infer Alpine tectonics. Moreover, the correlation of mafic rocks with former oceanic crust requires rock associations and compositions that are typical of an oceanic environment. The presence of rodingitized mafic dikes within meta-ultramafic rocks has been used as strong evidence for seafloor alteration prior to metamorphism (Bach & Klein, 2009; Coleman, 1963; Coleman et al., 1967; Frost, 1975). Additionally, oxygen isotopes can help to trace oceanic alteration of mafic rocks (e.g. Cartwright & Barnicoat, 1999; Früh-Green et al., 2001; Gregory & Taylor, 1981; Miller et al., 2001).

The peak metamorphic conditions of the mafic–ultramafic lenses of the Cima-Lunga unit and Southern Adula nappe (Alpe Arami, Cima di Gagnone and Monte Duria), where garnet peridotites occur, are in the range of 2.8–3.2 GPa and 750–840 °C (Brouwer et al., 2005; Ernst, 1981; Hermann et al., 2006; Nimis & Trommsdorff, 2001; Piccoli et al., 2022; Tumiati et al., 2018). These conditions are above the wet solidus of mafic rocks (Lambert & Wyllie, 1972) and metapelites (Hermann & Spandler, 2008). Indeed, in the metapelitic rocks of Cima di Gagnone, partial melting probably occurred during subduction (Piccoli et al., 2022). Therefore, when studying mafic–ultramafic lenses in the Southern Steep Belt of the Central Alps, special attention must be paid to identifying evidence of high-pressure (HP) partial melting.

In this study, we present new petrographic and geochronological data for the poorly studied mafic–ultramafic lenses from Val Cama and Valle di Moleno. We use petrography and zircon oxygen isotope composition to investigate the origin of these rock suites. The combination of zircon and rutile U–Pb dating and zircon, garnet and rutile geochemistry indicates that HP melting (~ 31 Ma) is closely followed by Barrovian amphibolite-facies metamorphism (~ 29 Ma). The new ages constrain a rapid transition from the final stages of subduction to the onset of collision in the Central Alps, implying a rapid exhumation process.

## Geological setting

**Fig. 1:**
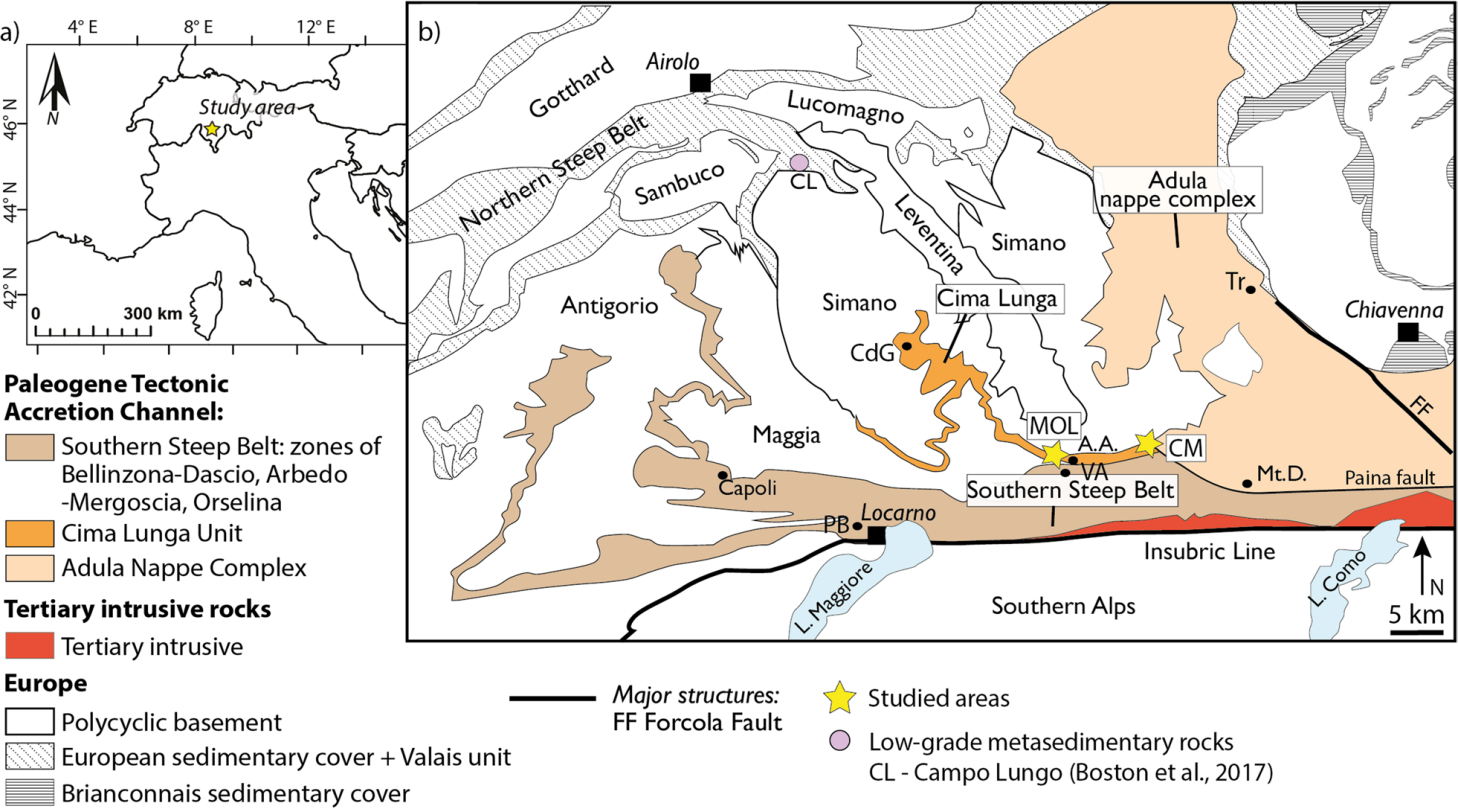
Tectonic map of the Central Alps modified from Piccoli et al. (2022). The studied areas are indicated by yellow stars: Valle di Moleno (MOL) and Val Cama (CM). A.A.: Alpe Arami; Mt.D.: Monte Duria; CdG: Cima di Gagnone; VA: Valle d’Arbedo; TR: Trescolmen; PB: Ponte Brolla

The Cima-Lunga unit and the Adula nappe (Fig. 1) are part of the Central Alps and belong to the Penninic nappe stack. They represent the distal European margin of the Piemont-Ligurian Ocean (Schmid et al., 1996), which was subducted and exhumed during the Alpine orogeny (Engi et al., 2001). The metamorphic grade in these units increases from north to south towards the Insubric Line, a major fault that separates the high-grade metamorphic rocks of the Central Alps from the low-grade metamorphic rocks of the Southern Alps. The Insubric Line is the result of the underthrusting of the European continental margin under the Apulian plate and the subsequent exhumation of the Central Alps during continent–continent collision (Schmid et al., 1987).

In the Cima-Lunga unit and the Adula nappe, eclogite-facies mafic–ultramafic lenses are preserved within the continental basement rocks composed of micaschists, paragneisses and orthogneisses metamorphosed at lower pressure, amphibolite facies metamorphic conditions (Brouwer et al., 2005; Dale & Holland, 2003; Evans & Trommsdorff, 1978; Pfiffner & Trommsdorff, 1998; Trommsdorff, 1990). The coexistence of low- and high-pressure rocks has been interpreted as a tectonic mélange (e.g. Berger et al., 2005; Brouwer et al., 2005; Trommsdorff, 1990). Other studies suggested that these rocks share a coherent metamorphic history during the Alpine subduction (e.g. Herwartz et al., 2011). A recent study proposed that the Cima-Lunga unit represents a reworked pre-Variscan metasedimentary basin (Tagliaferri et al., 2023).

In the northern Cima-Lunga unit, at Cima di Gagnone (Fig. 1) metamafic-ultramafic lenses consist of eclogites, garnet-amphibolites, metarodingites, chlorite-harzburgites and garnet-peridotites associated with carbonates, calcsilicate rocks and rare metapelites. These rocks record HP conditions of 2.8–3.2 GPa and temperatures of 750–800 °C (Nimis et al., 1999; Nimis & Trommsdorff, 2001; Piccoli et al., 2022; Scambelluri et al., 2014). In the southern part of the Cima-Lunga unit and the Adula nappe, at Alpe Arami and Monte Duria (Fig. 1), eclogites and metaperidotites record HP conditions of ca. 3.0 GPa and 800 °C and 3.0 GPa and 840 °C respectively (Brouwer et al., 2005; Ernst, 1981; Nimis & Trommsdorff, 2001; Tumiati et al., 2018). In the case of Alpe Arami garnet peridotite, there is a controversy about the peak pressure conditions, with studies suggesting e.g. up to 5.9 GPa (Paquin & Altherr, 2001). Geochronological data constrain peak pressure conditions in the northern and southern Cima-Lunga unit and southwestern Adula nappe to ~ 34–38 Ma (e.g. Brouwer et al., 2005; Gebauer et al., 1996; Hermann et al., 2006; Herwartz et al., 2011; Liati et al., 2009; Sandmann et al., 2014). Amphibolite-facies overprinting occurred after HP metamorphism and under conditions of 0.7–1.0 GPa and 600–650 °C at Cima di Gagnone (Corvò et al., 2021; Heinrich, 1982; Piccoli et al., 2022) and 0.7–1.4 GPa and 620–720 °C in the southern Cima-Lunga unit and Adula nappe (Brouwer et al., 2005; Dale & Holland, 2003; Engi et al., 1995; Todd & Engi, 1997). The amphibolite-facies overprint is attributed to the Barrovian metamorphism of the Lepontine Dome (Engi et al., 1995; Nagel et al., 2002; Todd & Engi, 1997) and is associated with the migmatisation of the Southern Steep Belt. This stage is dated to 32–22 Ma (e.g. Boston et al., 2017; Gebauer et al., 1996; Liati et al., 2009; Rubatto et al., 2009; Tagliaferri et al., 2023) and postdates nappe stacking (Pfiffner & Trommsdorff, 1998).

## Study area

The study areas of Valle di Moleno and Val Cama are part of the Cima-Lunga unit and the southern Adula nappe (Fig. 1) and are located in the Southern Steep Belt. All GPS coordinates of sample locations and visited outcrops as well as mineral assemblage proportions for the studied samples are given in Supplementary Table S1. The field observations are complemented with observations from the study of Vieira Duarte et al. (2023).

### Valle di Moleno

**Fig. 2:**
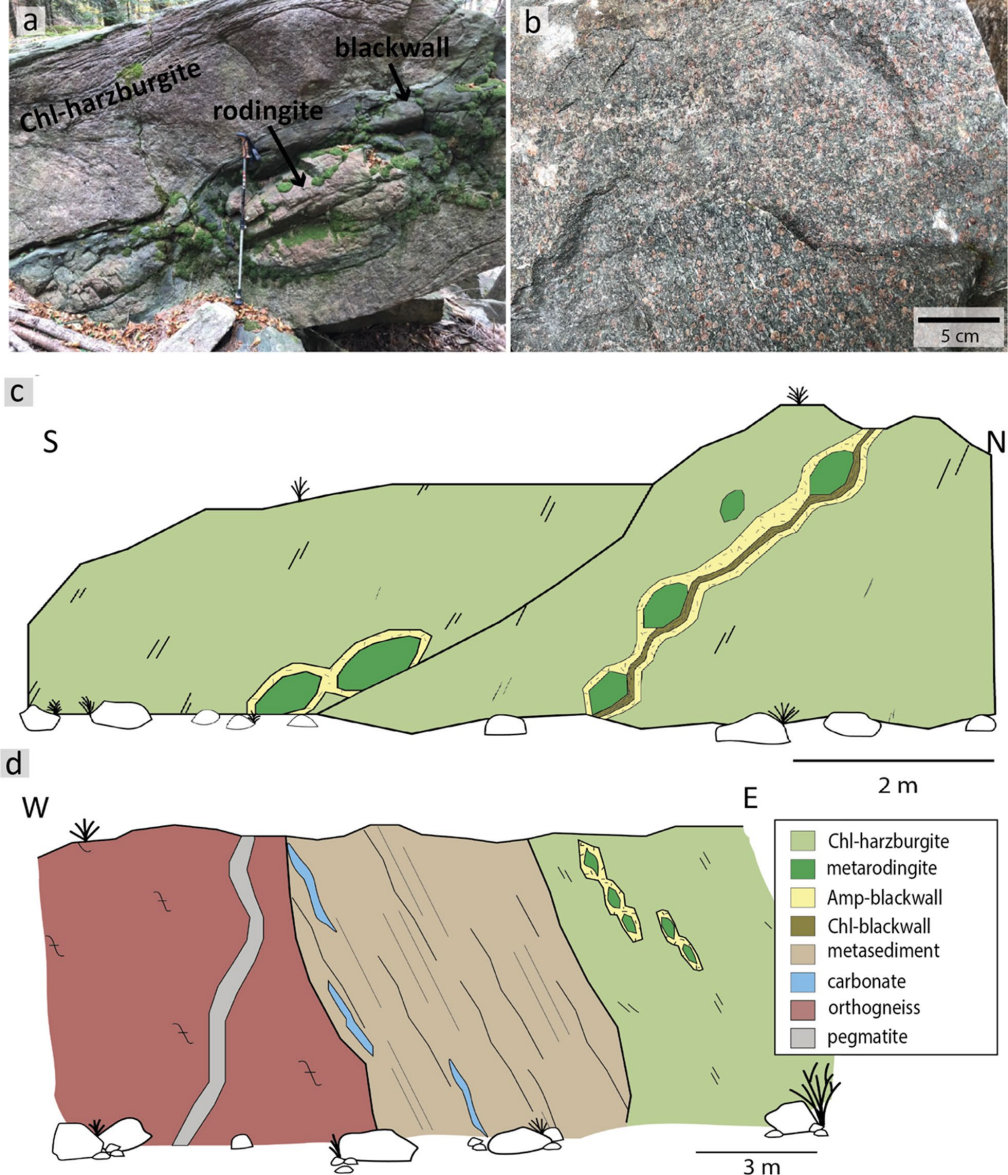
**a** Boudin of metarodingite with a blackwall in chlorite-harzburgite in Valle di Moleno (MOL). **b** Field image of retrogressed eclogite with large garnet porphyroblasts (here sample MOL21-2). **c** Sketch of field relations in the MOL locality showing metarodingites with blackwalls in Chl-harzburgite. **d** Sketch of field relations of the outcrop in Val Cama. Gneiss is adjacent to metasediments and ultramafic rocks that host boudins of metarodingites

Large metamafic-ultramafic bodies and migmatised gneisses occur on the steep flanks of Monte Mottascio in Valle di Moleno (Ticino, Switzerland) and crop out along streambeds on the north-western flank of Monte Mottascio and below the Capanna Gariss. The ultramafic rocks are usually chlorite-harzburgites with local retrograde alteration features. They consist of orthopyroxene, olivine, chlorite, carbonate and minor talc with mm to cm-sized magnetite aggregates (MOL21-3 & MOL21-7a, Table S1). Some ultramafic bodies are foliated with aligned chlorite grains and magnetite aggregates, while others are massive or show a spinifex texture with spinifex orthopyroxene. Chlorite pseudomorphs after garnet have been observed in a sample of chlorite-peridotite (Camenzind, pers.comm.). The ultramafic rocks host lenses of garnet-amphibolites or metarodingites, which occasionally show an amphibole or chlorite blackwall of up to 30 cm (Fig. 2a and c). Lenses of garnet-amphibolites and metarodingites have also been observed in gneissic rocks on the northern flank of Monte Mottascio. Samples MOL20-1 and MOL21-2 are retrogressed eclogites (Fig. 2b) and were collected as boulders (ca. 30 × 40 cm and 25 × 35 cm respectively) from a stream bed on the northern flank of Monte Mottascio (MOL21-2) and from a stream bed in the village of Moleno (MOL20-1). Based on the local topography and where the sample MOL21-2 was found, it could be possible that an eclogite lens is located on the steep, northern flank of Monte Mottascio.

### Val Cama

The studied outcrop in Val Cama (Fig. 2d, Graubünden, Switzerland) is a 10 × 15 m steep wall below Piz Croch (ca. 1100 m a.s.l.). It consists of orthogneisses with cross-cutting pegmatites adjacent to metasedimentary and ultramafic rocks. The foliation in the orthogneiss is concordant to the contact with the metasediments. The metasediments are coarse-grained garnet-diopside calcsilicate rocks, banded and intercalated with metacarbonate lenses. At the contact with the ultramafic rocks, the metasediments are carbonate-free. Ultramafic rocks from this locality are described by Hofmann and Bühl (1982) as chlorite-metaperidotite consisting of enstatite, olivine, chlorite, and magnetite. Vieira Duarte et al. (2023) describe the ultramafic rocks from other localities in Val Cama as (hematite)-magnetite chlorite-peridotites and chromite-chlorite-peridotites based on their peak temperature mineral assemblage. They consist of variable amounts of olivine, orthopyroxene and chlorite and occasionally show retrograde Mg-anthophyllite. The ultramafic rocks of the visited outcrop host boudinaged layers of metarodingites, which show an amphibole blackwall at the contact. Samples CM20-8 (garnet-diopside fels) and CM20-12 (metarodingite) were collected from this outcrop, while CM20-2 (garnet amphibolite) and CM20-6 (metarodingite) were collected from the Rià de Cama riverbed, a few hundred meters away.

## Methods

### Microscopy and imaging

A petrographic microscope and a Zeiss Evo 50 scanning electron microscope (SEM, Institute of Geological Sciences, University of Bern) were used for the petrographic examination of thin sections. SEM backscattered electron (BSE) images of minerals and textures in thin sections and epoxy mounts were obtained using a working distance of 9.5–10 mm, an acceleration voltage of 15–20 kV and a current of 1 nA. In addition, charge contrast (CC) imaging was used to recognize zircon textures (Griffin, 2000) and was performed in the variable pressure mode using a working distance of 9.5–10 mm, an acceleration voltage of 15–20 kV, and a beam current of 1 nA.

### EPMA

The JEOL JXA 8200 Superprobe (Institute of Geological Sciences, University of Bern) was used to determine the mineral chemistry using wavelength dispersive spectrometers (WDS). Point analyses of silicates, rutile, and spinel were obtained as well as chemical maps of garnet. The point analyses were measured with an acceleration voltage of 15 kV and a beam current of 20 nA. Counting times on the peak were 20 s and 10 s for the background on either side of the peak. The maps were measured with an acceleration voltage of 15 kV and a beam current of 100 nA, with dwell times of 90–100 ms. Natural and synthetic standards were used for the calibration of the following element oxides: SiO_2_ (wollastonite), Al_2_O_3_ (anorthite), CaO (anorthite), Na_2_O (albite), K_2_O (orthoclase), FeO (olivine), MnO (tephroite), MgO (olivine), TiO_2_ (rutile), and P_2_O_5_ (apatite). Chemical maps were processed in XMapTools using point analyses to calibrate the maps (Lanari et al., 2014). Mineral analyses are given in Supplementary Table 2.

### Mineral separation and epoxy mount

Samples were fragmented using a Selfrag apparatus (Institute of Geological Sciences, University of Bern), which uses high-voltage pulses to split mineral grains along their grain boundaries. The material was then wet-sieved to get a grain fraction of 250–64 µm. Zircon and rutile were magnetically separated from the 250–64 µm fraction using a ferromagnetic Franz-separator (I > 1.2 A). Density separation was performed on the non-magnetic fraction using the density liquid DI (3.32 g/cm^3^). Hand-picked, individual grains of zircon and rutile were mounted in epoxy resin, ground with sandpaper and polished to 1 μm with diamond paste.

### LA-ICP-MS

A laser ablation inductively coupled plasma mass spectrometer (LA-ICP-MS) was used to determine isotopes for the U–Pb dating of zircon and rutile as well as for major, minor, and trace elements of zircon, garnet, epidote/clinozoisite and rutile. Analyses were performed at the Institute of Geological Sciences, University of Bern, using the Resolution LA system coupled to an Agilent 7900 ICPMS.

#### U–Pb dating of zircon

An energy density of 4 J/cm^2^, a repetition rate of 5 Hz and spot sizes of 24 µm (MOL20-1), 30 µm (CM20-6 and CM20-12) and 38 µm (MOL 21-2, CM20-2, and CM20-8) were used. A gas mixture of N_2_-He was used for aerosol transport. Masses ^49^Ti, ^202^Hg, ^204^Pb, ^206^Pb, ^207^Pb, ^208^Pb, ^232^Th, and ^238^U were measured. The dwell times ranged from 20 to 40 ms. The analyses were performed in two sessions. The Temora zircon (416.75 ± 0.24 Ma, Black et al., 2003) was used as the primary standard and the Plešovice zircon (337.13 ± 0.37 Ma, Sláma et al., 2008) and 91,500 zircon (1065 Ma, Wiedenbeck et al., 1995) were used as secondary standards. The measured ages for Plešovice and 91,500 are within 1% of the reference value for session I (Plesovice: 340.5 ± 1.6 Ma, 91500: 1073.1 ± 5.8 Ma); in session II 91500 overlaps with the reference age (1065.7 ± 6.2 Ma). Data processing was performed with Iolite (Hellstrom et al., 2008; Paton et al., 2011), applying a common Pb correction (correction for ^207^Pb) and using the UcomPbine DRS whenever appropriate (detected common Pb). Average ages are obtained by regression on a Tera-Wasserburg (TW) plot for uncorrected ratios, assuming a present-day common Pb composition (Stacey & Kramers, 1975). In most samples, the low dispersion of the analyses does not allow using a free regression. However, in each sample, there are analyses that overlap with the reverse concordia, and thus the age would not change within the uncertainty by assuming a different common Pb composition (see the example below for zircon rims from sample CM20-8*)*. Ages were calculated and plotted using Isoplot for Excel (Ludwig, 2003) and average ages are given at a 95% confidence level. Supplementary Table 3 lists the values used for the age calculations and for the TW plots.

#### U–Pb dating of rutile

U–Pb dating of rutile was performed using an energy density of 3 J/cm^2^, a repetition rate of 5 Hz and a laser beam diameter of 54 µm. The masses ^206^Pb, ^207^Pb, ^208^Pb, ^232^Th, ^235^U, and ^238^U were measured. Rutile R10 (1095.2 ± 4.7 Ma, Luvizotto et al., 2009) was used as the primary reference material and rutile R19 (489.5 ± 0.9 Ma, Zack et al., 2011) as the secondary reference material. The measured ages for the R10 and R19 yield ages that lie within the uncertainty of the reference values (R10: 1090.1 ± 4.5 Ma, R19: 488.2 ± 5.0 Ma). Data processing was performed using the Iolite software (Hellstrom et al., 2008), and age calculation and plotting were done with Isoplot for Excel (Ludwig, 2003). Average ages were obtained by data regression in the TW diagram using ratios uncorrected for common Pb (see details on common Pb composition in the zircon method). Correction for common ^208^Pb was performed whenever relevant using the VizualAge UcomPbine data reduction and ^208^Pb (no Th) correction to obtain dates on individual analyses. The analyses that have been used for age calculation and plotted in the TW diagram are given in Supplementary Table 4.

#### Trace element analyses

The determination of major-, minor- and trace elements in zircon, garnet, epidote minerals and rutile was done using a frequency of 5–9 Hz, energy densities of 3–4 J/cm^2^, and an N_2_-He gas mixture for aerosol transport. Dwell times were between 10 and 30 ms and spot sizes were between 30 and 54 µm (see Supplementary Table 5 for details of the measurement conditions and results). Instrument conditions were optimized to achieve oxide production of Th/ThO < 0.2%. The measurements have been performed in six different sessions using the GSD-1 g glass (Jochum et al., 2011) or NIST612 as the primary standard and NIST612 or NIST610 (Jochum et al., 2005) as the secondary standard (the standards used for the respective measurements are listed in the Supplementary Table 5). The REE analyses of the glass standards are within 15% variation of the reference values, Ti is within 10% and Zr is within 12% of the reference values. Zircon and rutile were measured in separate sessions. Stoichiometric abundances of Si or Al (silicates) and Ti (rutile) were used for internal calibration. Data evaluation was performed with the Iolite software (Hellstrom et al., 2008; Paton et al., 2011). Detection limits were calculated in Iolite following Pettke et al. (2012).

### SIMS

Oxygen isotopes were measured in zircon using the CAMECA SIMS 1280HR high-resolution ion microprobe at the SwissSIMS facility (University of Lausanne, Switzerland), following the conditions of Seitz et al. (2017). Measurements were performed with a Cs primary Gaussian beam (10 kV), resulting in a spot size of 15–20 μm, and a beam current of ~ 1.75 nA. The obtained ^18^O/^16^O ratios were converted to δ^18^O in ‰ relative to the Vienna Standard Mean Ocean Water (VSMOW, Supplementary Table 6). Oxygen isotopes were determined in three sessions using the 91,500 (9.86 ± 0.11 ‰, Wiedenbeck et al., 2004) and Temora zircon (8.20 ± 0.02 ‰ Black et al., 2004) as primary and secondary reference materials, respectively.

## Results

### Petrography and mineral assemblages

The description below focuses on major-, minor- and accessory mineral phases that are important for the interpretation of the multiple stages recorded by the samples (Table 1 and S1).

**Table 1:** Overview of samples of this study with their major-, minor- and accessory minerals, microtextures, and interpreted HP and HT assemblages

Sample	Rock type	Major minerals	Minor minerals	Accessory minerals	Micro textures	HP Assemblages	HT Assemblages
MOL20-1	Retrogressed clinozoisite eclogite	Grt, Czo, Amp, Pl	Omp, Qtz	Zr	Amp-Pl-sympl^1^	Grt + Omp + Czo + Rt + Qtz + Zr	Amp + Pl
MOL21-2	Retrogressed clinozoisite-kyanite eclogite	Grt, Amp, Czo, Ky, Pl	Ky, Qtz	Zr, Tnt	Amp-Pl-sympl^1^, Pl-Spi-sympl^2^	Grt + Czo + Ky + Rt + Qtz + Zr	Amp + Pl
CM20-2	Garnet amphibolite	Amp, Grt	Pl, Rt	Zr	–	–	Amp + Pl
CM20-6	Metarodingite	Grt, Cpx, Amp	Ep	Zr	Amp replacing Cpx rims	–	–
CM20-12	Metarodingite	Grt, Cpx	Mt, Spi, Ap	Zr	–	–	–
CM20-8	Metasediment	Grt, Cpx	Cc, Tnt, Ap	Zr	–	–	–

^1^Amp-Pl-symplectite around garnet and in the matrix

^2^Pl-Spi-symplectite around kyanite

**Fig. 3:**
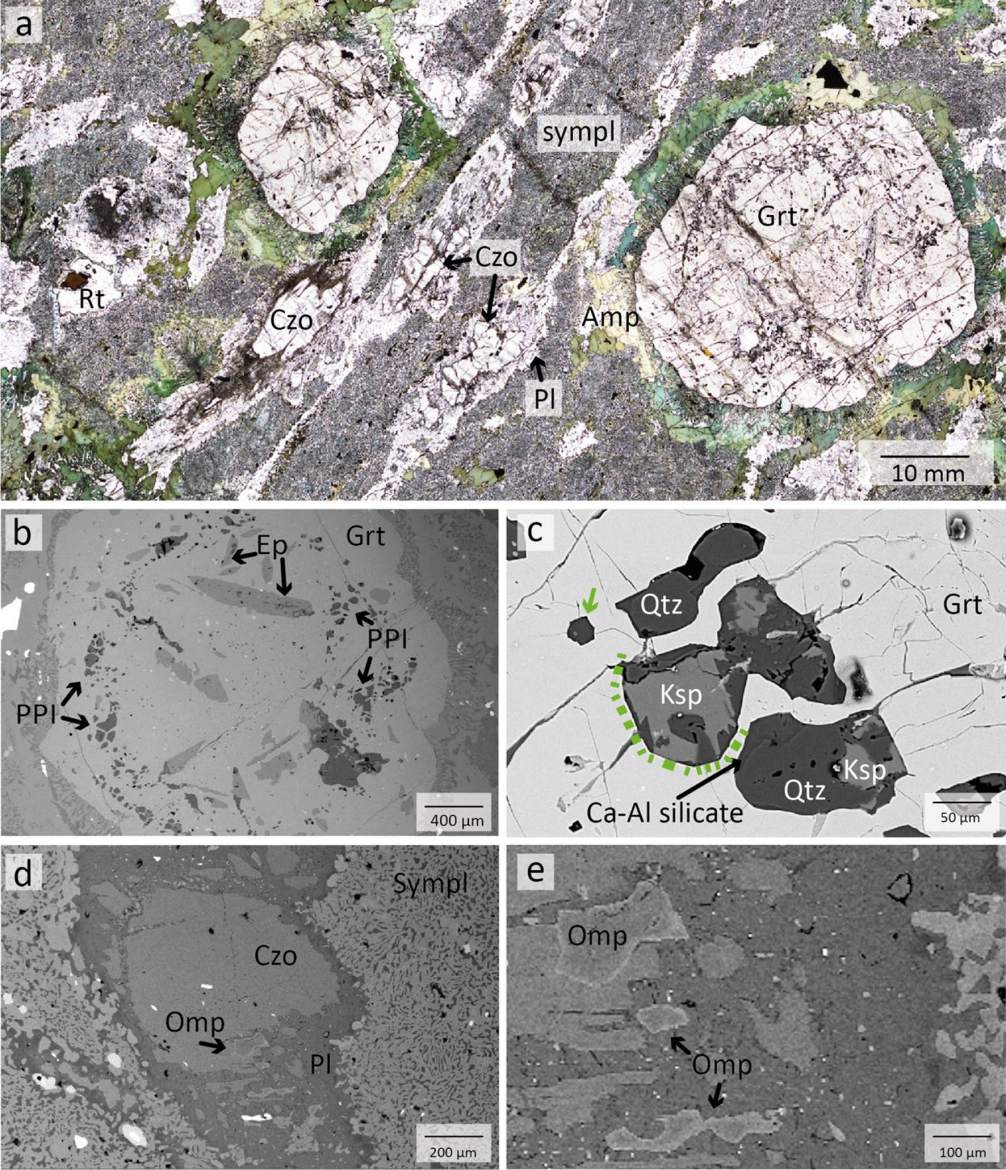
**a** Thin section image of retrogressed clinozoisite eclogite MOL20-1 showing large garnet porphyroblasts and major minerals embedded in a fine-grained matrix of amphibole-plagioclase symplectite. **b** BSE image showing the distribution of inclusions in a garnet porphyroblast. Polyphase inclusions (PPI) are concentrically distributed in the garnet mantle. **c** Polyphase inclusions of Qtz, K-fsp, Pl, and a Ca-Al silicate in the garnet mantle. The green arrow and dashed line indicate negative crystal shapes. **d** Clinozoisite surrounded by plagioclase in matrix symplectite. **e** Detail of image **d** showing relicts of omphacite in plagioclase

**Fig. 4:**
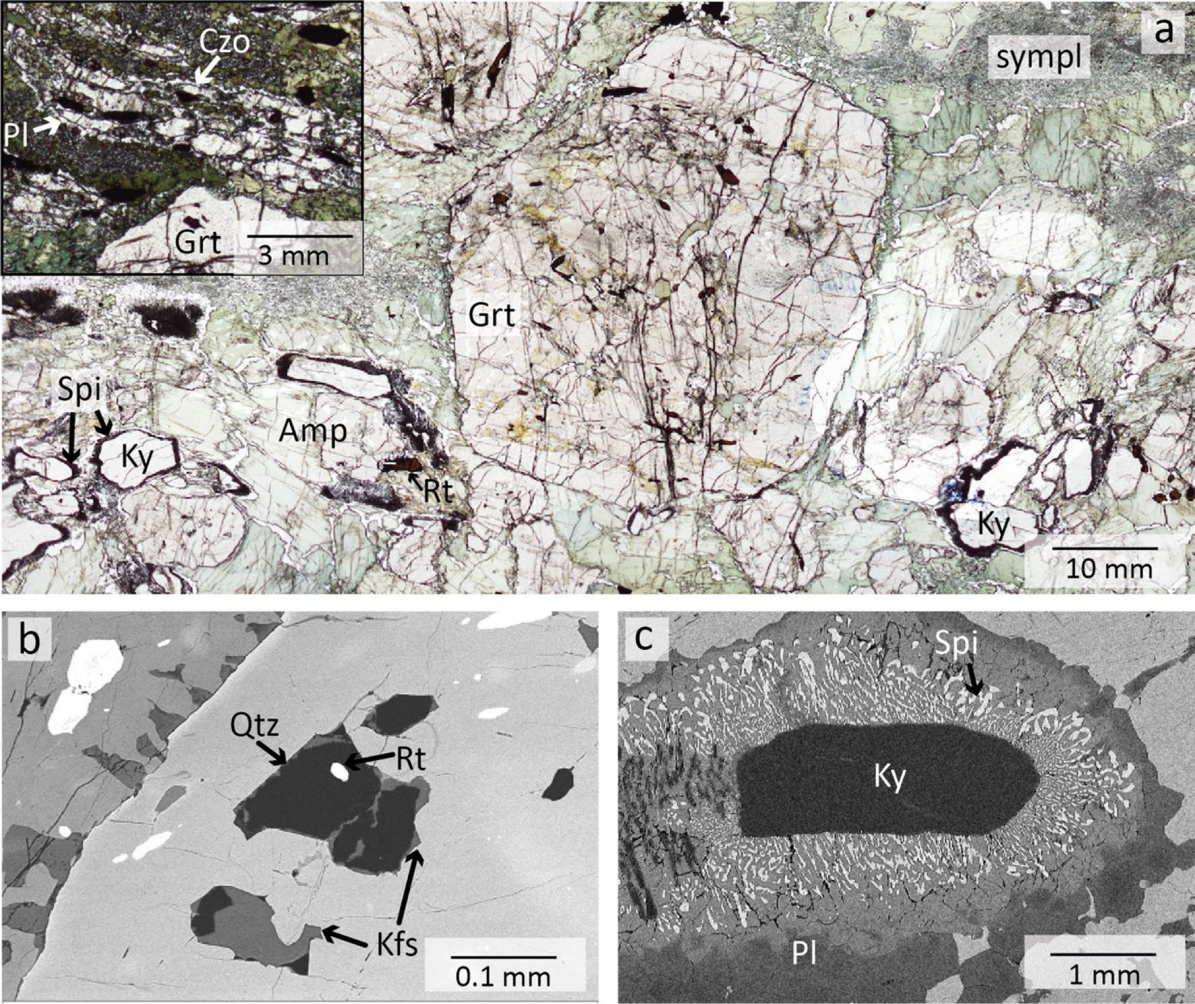
Thin section images of retrogressed clinozoisite-kyanite eclogite (MOL21-2) **a** Overview showing a large garnet porphyroblast surrounded by amphibole. The kyanite is surrounded by a spinel-anorthite symplectite (black). The upper left image shows clinozoisite grains surrounded by plagioclase. **b** BSE image of polyphase inclusions of quartz, K-feldspar, and rutile in garnet mantle. **c** BSE image of kyanite surrounded by a spinel-anorthite symplectite and plagioclase

*Retrogressed eclogites* (MOL20-1 & MOL21-2, Figs. 3 and 4) contain garnet, amphibole, clinozoisite/epidote, kyanite (only in MOL21-2) and plagioclase as major phases. Minor phases are quartz, omphacite (only in MOL20-1), rutile, and ilmenite. Zircon and titanite occur as accessory phases. Garnet occurs as centimetre-sized porphyroblasts, many of which are altered and partially or completely replaced by amphibole (Fig. 3a). Garnet can be divided into core, mantle and rim domains based on its composition (Sect. 5.2) and inclusion assemblages. Garnet cores contain both large epidote and clinozoisite grains (up to 0.8 mm) as well as micrometre-sized rutile, quartz, plagioclase, and carbonates. No coesite or radial cracks surrounding quartz inclusions could be observed in garnet cores. Garnet mantle contains large clinozoisite/epidote, rutile, zircon, and polyphase inclusions. Some garnet rims contain epidote and plagioclase. Polyphase inclusions (Figs. 3c and 4b) in the garnet mantle show varying sizes of ~ 15 to 200 µm and a negative crystal shape or irregular shapes. Mineral phases that could be identified in these inclusions comprise K-feldspar, quartz, albitic plagioclase and a Ca-Al silicate, which show sometimes intergrowths. One polyphase inclusion contains rutile.

Clinozoisite is partly retrogressed and surrounded by plagioclase (Fig. 3a and d). Omphacite is found as micrometre-sized relics, between clinozoisite grains or in the matrix (Fig. 3e). Amphibole replaces garnet and occurs as large grains around garnet (Fig. 3a) or in the matrix (Fig. 4a). In some parts of sample MOL21-2, large amphibole grains are hypidiomorphic and the space between single amphibole grains of garnet are filled by plagioclase. In some parts these amphibole grains show a sharp mineral boundary between single grains. This may indicate a previous equilibrium texture and a possible replacement of former omphacite by amphibole and plagioclase (Fig. 4a). Both retrogressed eclogites show amphibole-plagioclase symplectites, one replacing garnet and the other in the matrix probably replacing former omphacite (Figs. 3a and 4a). Sample MOL21-2 also shows a spinel-anorthite symplectite around kyanite (Fig. 4c). Rutile occurs as single grains (up to 1 mm) or as intergrowth with ilmenite which is partly rimmed by titanite.

**Fig. 5:**
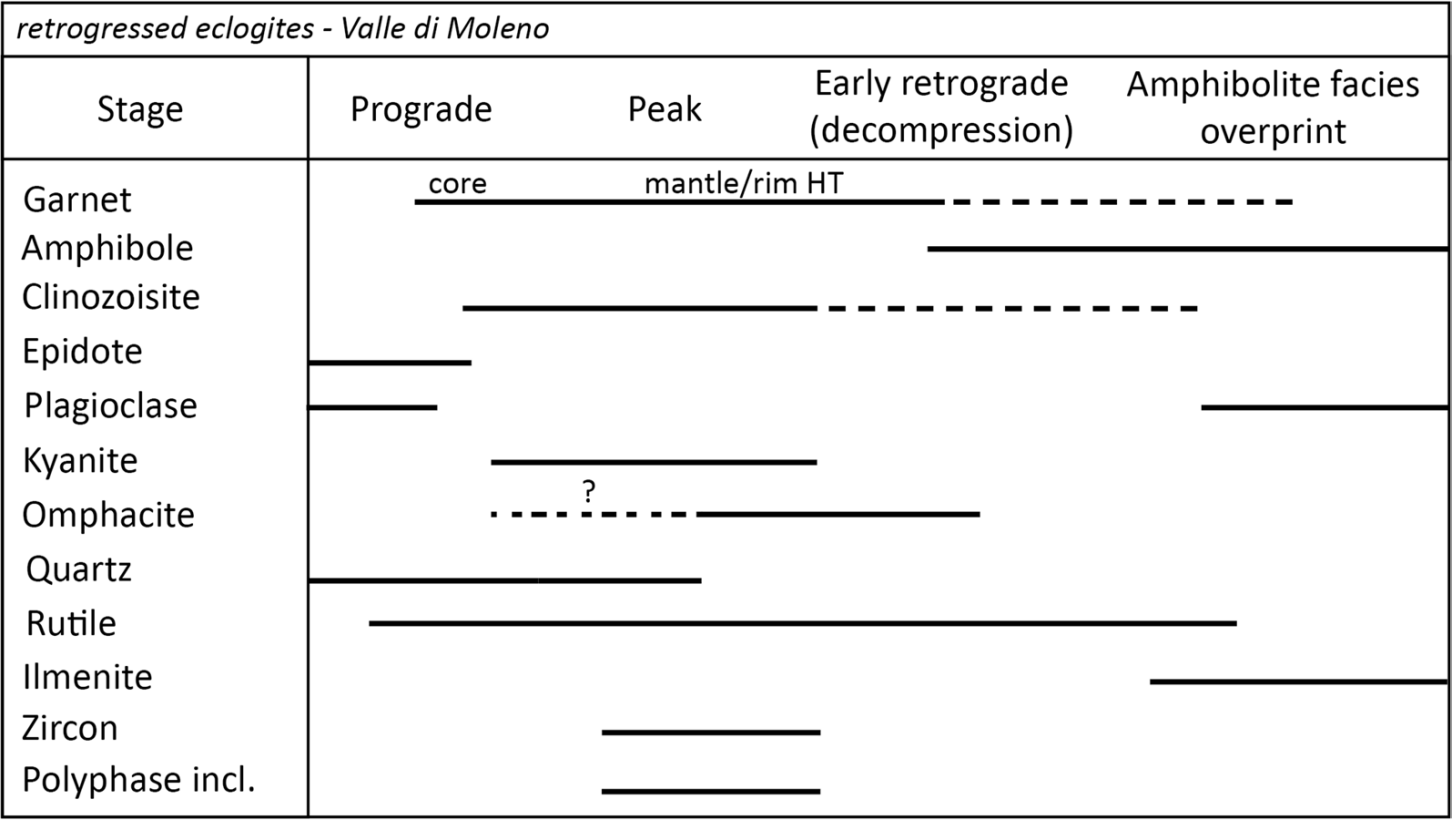
Paragenesis table of MOL retrogressed eclogites summarizing the main petrographic observations with minerals classified according to the metamorphic stages. Dashed lines indicate the possible stability of certain minerals

Based on the above petrographic observations we interpret Grt + Czo + Omp + Qtz + Rt + Zr (MOL20-1) and Grt + Ky + Czo + Rt + Zr + Qtz (MOL21-2) as the peak HP paragenesis (see also Fig. 5). Relic omphacite was observed in the matrix and partly replacing matrix clinozoisite, but not as inclusions in garnet. Therefore, we interpret that omphacite was in equilibrium with the garnet rim. The amphibole-plagioclase symplectite around garnet and in the matrix as well as the anorthite-spinel symplectite around kyanite indicate decompression after high-pressure metamorphism. Large amphibole grains around and replacing garnet and in the matrix coexist with plagioclase, indicating an amphibolite facies re-equilibrium. During this stage matrix clinozoisite is replaced by anorthite.

**Fig. 6:**
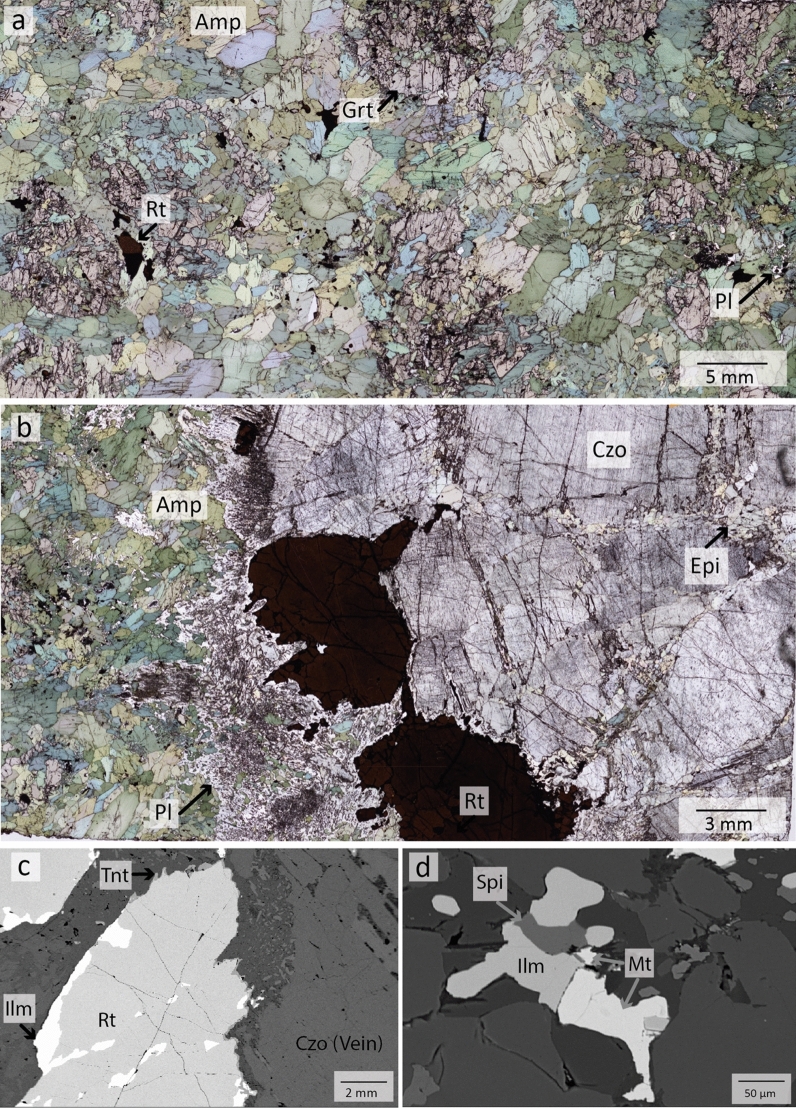
Thin section images of garnet amphibolite CM20-2 **a** Overview showing large, decomposed garnet porphyroblasts in a matrix of amphibole grains; plagioclase, rutile, ilmenite, magnetite and spinel occur in the matrix. **b** The rutile-clinozoisite vein (right side) with plagioclase between vein and host rock (left). **c** BSE image of vein rutile which is partly intergrown with ilmenite and shows a micrometre-sized seam of titanite. **d** BSE image of magnetite-ilmenite-spinel intergrowth

The major minerals in the garnet amphibolite CM20-2 (Fig. 6a and b) are amphibole and garnet. Plagioclase, quartz, magnetite, spinel, rutile, and titanite are minor phases and zircon occurs as an accessory phase. The garnet amphibolite is cut by a vein consisting of coarse-grained clinozoisite and rutile (up to 5 mm, Fig. 6b). Garnet is found as centimetre-sized porphyroblasts, which are strongly altered and replaced by amphibole (Fig. 6a). Amphibole occurs as hypidiomorphic to xenomorphic grains that form the matrix and show colours of yellow-green, green, green–blue and blue (Fig. 6a and b). Minor plagioclase occurs between the vein and host rock and in the matrix (Fig. 6b). Matrix rutile is found as single grains or is intergrown with ilmenite. Magnetite, ilmenite and spinel are found as intergrowths in the matrix. (Fig. 6d). Vein rutile is intergrown with ilmenite and partly shows a micrometre-sized seam of titanite (Fig. 6c).

We interpret that this sample underwent amphibolite-facies metamorphism represented by the assemblage of amphibole and (minor) plagioclase coexisting with garnet and rutile. This sample shows no petrographically evidence of prior eclogite facies metamorphism.

**Fig. 7:**
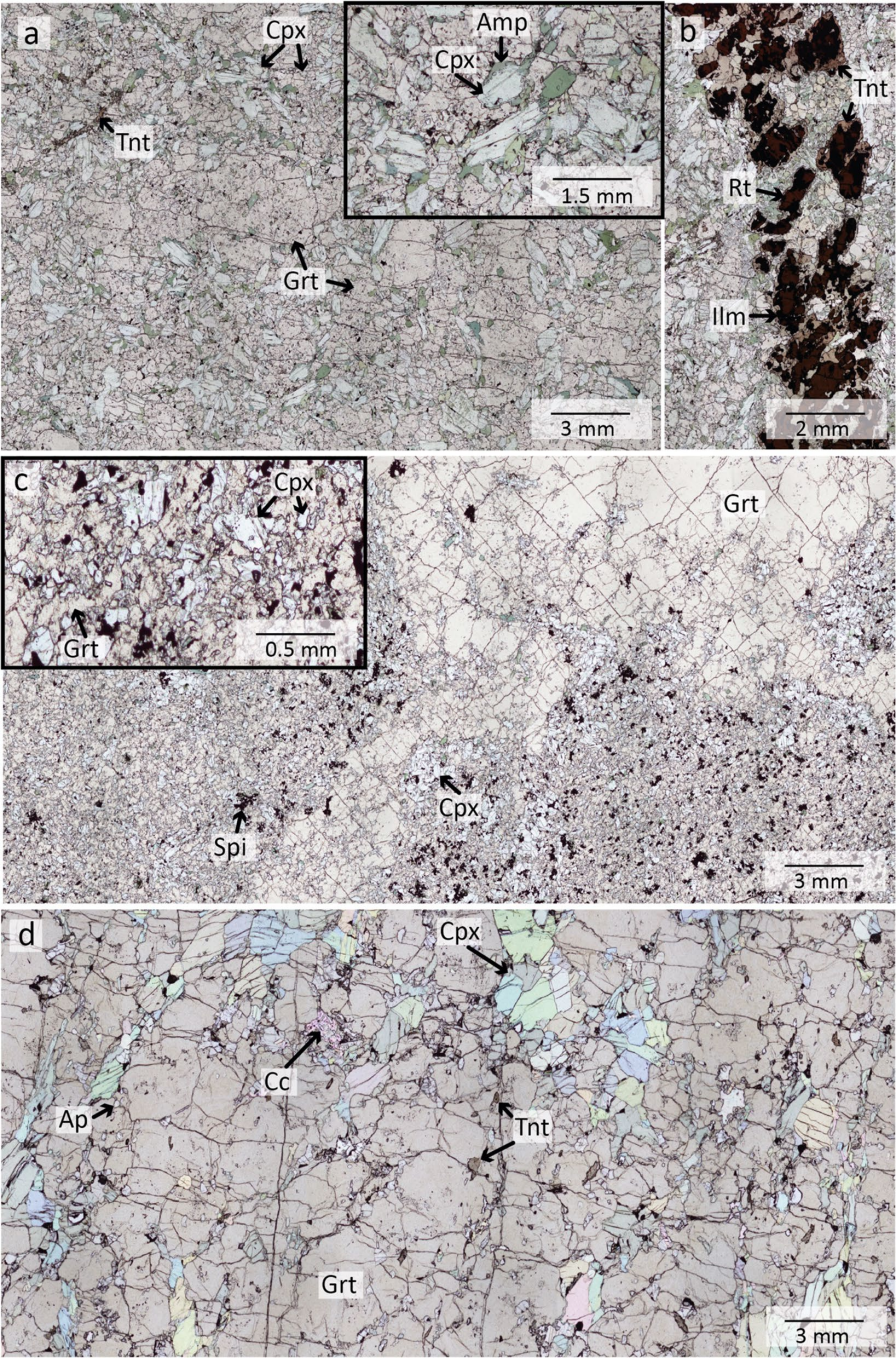
Thin section images of metarodingites CM20-6 and CM20-12 and metasediment CM20-8. **a** Overview of CM20-6 showing fine-grained matrix of garnet and clinopyroxene. The image in the upper right corner shows clinopyroxene rims replaced by amphibole. **b** Vein in sample CM20-6 containing rutile intergrown with ilmenite and rimmed by titanite. **c** Overview of CM20-12 showing massive garnet aggregates and the fine-grained matrix of garnet, clinopyroxene and spinel. The upper left image shows the matrix enlarged. **d** Overview image of CM20-8 showing clinopyroxene, carbonates, apatite, and titanite between massive garnet

*Metarodingites CM20-6 & CM20-12* (Fig. 7a–c) have comparable mineralogy with garnet, clinopyroxene, and amphibole as major minerals. Minor phases of sample CM20-6 comprise ilmenite and titanite whereas CM20-12 shows magnetite, spinel, and apatite as minor phases. Zircon occurs as an accessory phase in both samples. CM 20-6 is cut by a vein consisting of large rutile grains (1–4 mm, Fig. 7b) which are intergrown with ilmenite and rimmed by titanite.

Sample CM20-6 is a fine-grained rodingite with abundant garnet and clinopyroxene in the matrix. Clinopyroxene rims are replaced by amphibole (Fig. 7a). Sample CM20-12 is microtexturally different, with large garnet aggregates in a fine-grained matrix of garnet, clinopyroxene, and amphibole (Fig. 7c).

*Metasediment CM20-8* (Fig. 7d) consists of garnet, clinopyroxene and epidote as major phases. Apatite, titanite, and calcite occur as minor phases and zircon is an accessory. Garnet occurs as aggregates and xenomorphic diopside, epidote, calcite, and large apatite grains (~ 1 mm) fill the space between these massive aggregates.

### Mineral chemistry

The following description focuses on the composition of the major rock-forming minerals (Supplementary Table S2). Compositions of minor phases such as kyanite, magnetite, spinel, ilmenite, apatite, and carbonate are shown in the table but are not described below.

#### Garnet

**Fig. 8:**
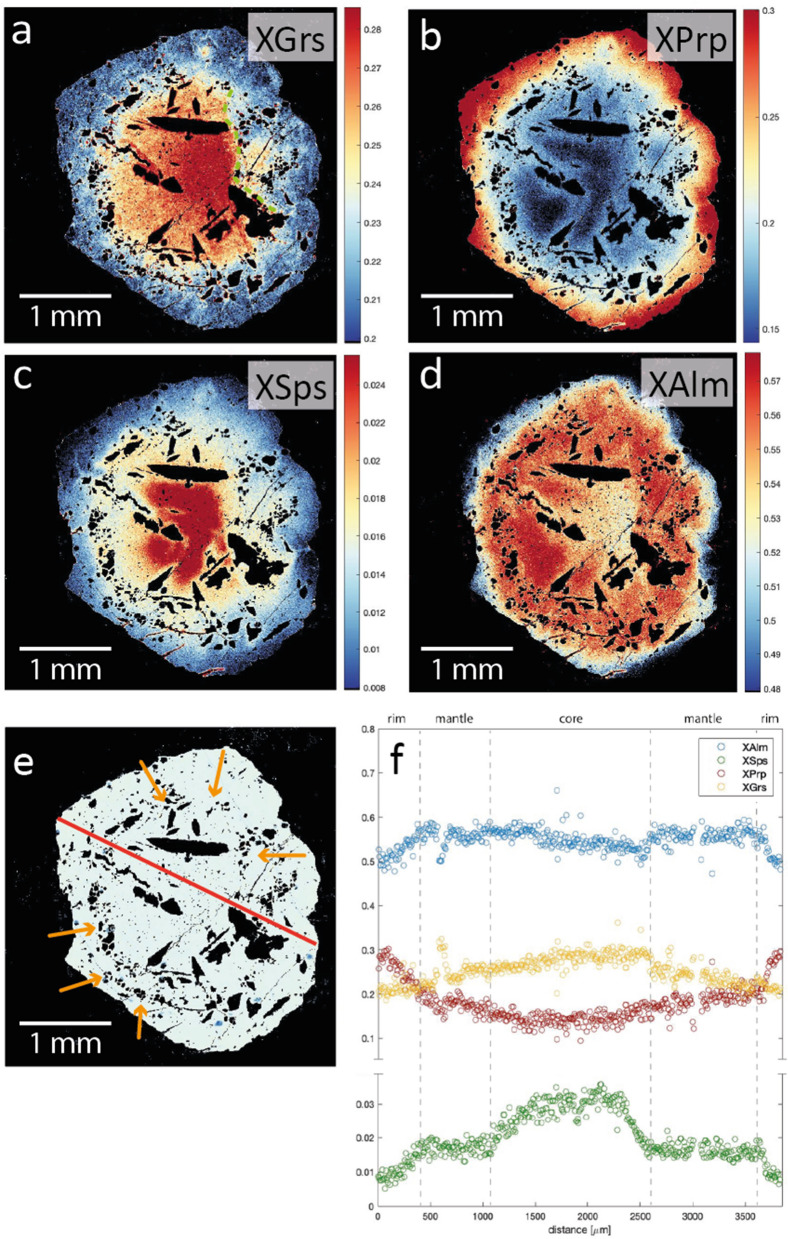
Garnet endmember maps of retrogressed clinozoisite eclogite MOL20-1: **a** X_Grs_, **b** X_Prp_, **c** X_Sps_, **d** X_Alm_. Dashed green line in **a** indicates the truncated garnet zonation. **e** BSE image of the same garnet with orange arrows indicating polyphase inclusions. **f** Garnet composition zoning along a rim-core-rim profile for the same garnet as in **a**–**e** extracted from garnet endmember maps

**Fig. 9:**
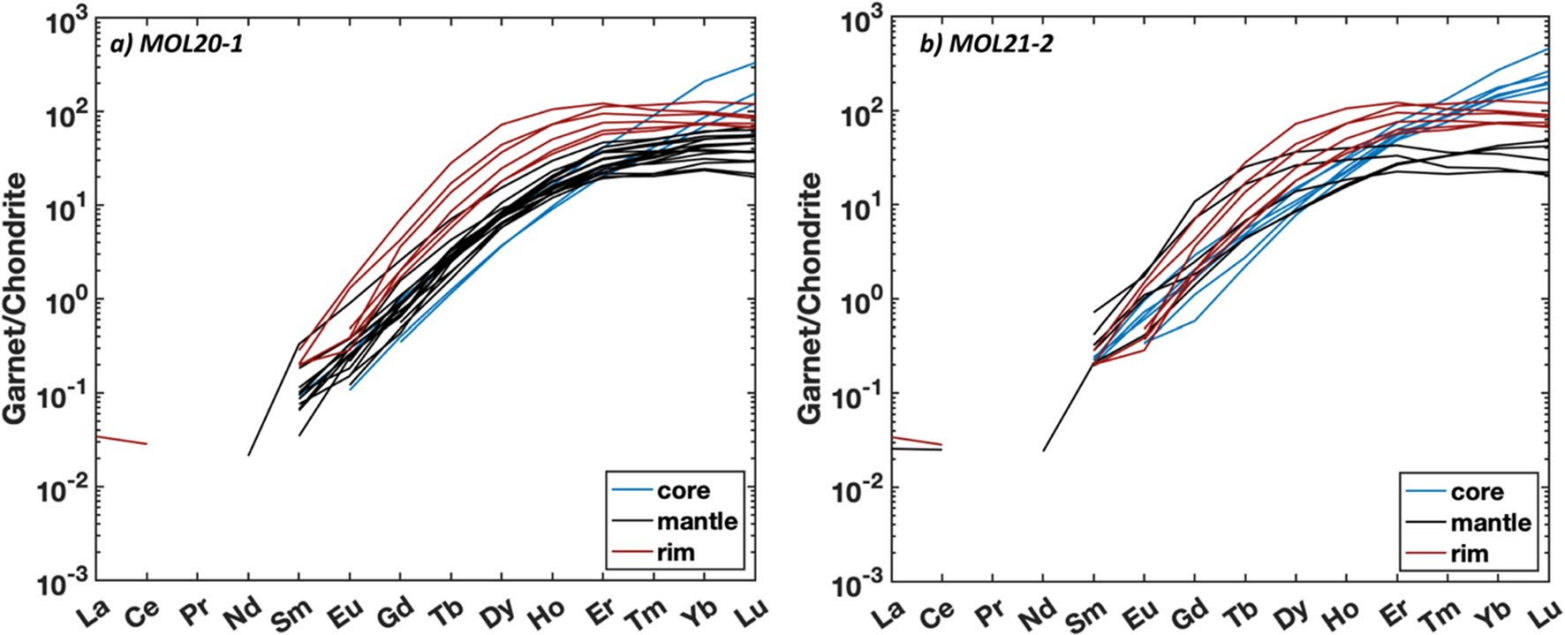
Chondrite normalized (Boynton, 1984) REE pattern of garnet core, mantle, and rim of retrogressed eclogites a) MOL20-1 and b) MOL21-2

Garnets of both retrogressed eclogites from Valle di Moleno show a similar internal zonation pattern of major elements for core, mantle, and rim (Fig. 8 and Supplementary File 1, Fig. S1). In general, garnet in sample MOL21-2 shows a slightly higher Mg-content than garnet of sample MOL20-1 (maximum X_Prp_ is 0.42 and 0.39, respectively). Garnet cores of sample MOL20-1 have X_Sps_ (0.02–0.04) and show a higher X_Grs_ (0.28) compared to garnet mantle (X_Grs_ = 0.24) and rim (X_Grs_ = 0.21). X_Alm_ increases from core (X_Alm_ = 0.46) to mantle (X_Alm_ up to 0.57) and decreases in the rim (X_Alm_ = 0.48). The X_Prp_ content increases from core (X_Prp_ = 0.16) towards the rim (X_Prp_ = 0.30). Garnet in sample MOL21-2 shows resorption rims (~ 30–50 µm) with X_Sps_ up to 0.03. The compositional maps (Fig. 8a–d) show that the garnet zoning in sample MOL20-1 is truncated (best recognized in the X_Grs_ map, Fig. 8a)_._ Along this disturbance, polyphase inclusions of quartz, albite, K-feldspar, and a hydrous Ca-Al–silicate phase are distributed (Fig. 8e). Notably, the X_Prp_ is significantly increasing towards the rim after the occurrence of the polyphase inclusions (Fig. 8b). The REE patterns (Fig. 9) of garnet cores for samples MOL20-1 and MOL21-2 are characterized by a steep slope for MREE and HREE (Dy_N_/Lu_N_ = 0.02–0.06), whereas the mantle and rim show a flat HREE pattern (Dy_N_/Lu_N_ = 0.12–1.27).

The retrogressed garnet porphyroblasts of the garnet amphibolite CM20-2 show variability in chemical composition (Supplementary Table S2.1). Due to strong alteration of garnet in this sample, it is not possible to link the composition to specific garnet domains. The composition varies for X_Alm_ from 0.35 to 0.51, for X_Grs_ from 0.21 to 0.33, for X_Prp_ from 0.21 to 0.31 and for X_Sps_ from 0.01 to 0.03. Garnet of the metarodingite CM20-6 shows an average composition of X_Alm_ = 0.48, X_Grs_ = 0.22, X_Prp_ = 0.28 and X_Sps_ = 0.01, while in sample CM20-12, garnet has higher X_Grs_ = 0.48 component, lower X_Alm_ = 0.30 and X_Prp_ = 0.21 and a similar X_Sps_ = 0.01 component. Massive garnet aggregates of the calcsilicate rock CM20-8 show a composition rich in X_Grs_ = 0.85–0.76 and low in X_Alm_ = 0.11–0.05 and X_And_ = 0.15–0.07. X_Sps_ varies from 0.02–0.01. Mg is under the detection limit.

#### Epidote/clinozoisite

**Fig. 10:**
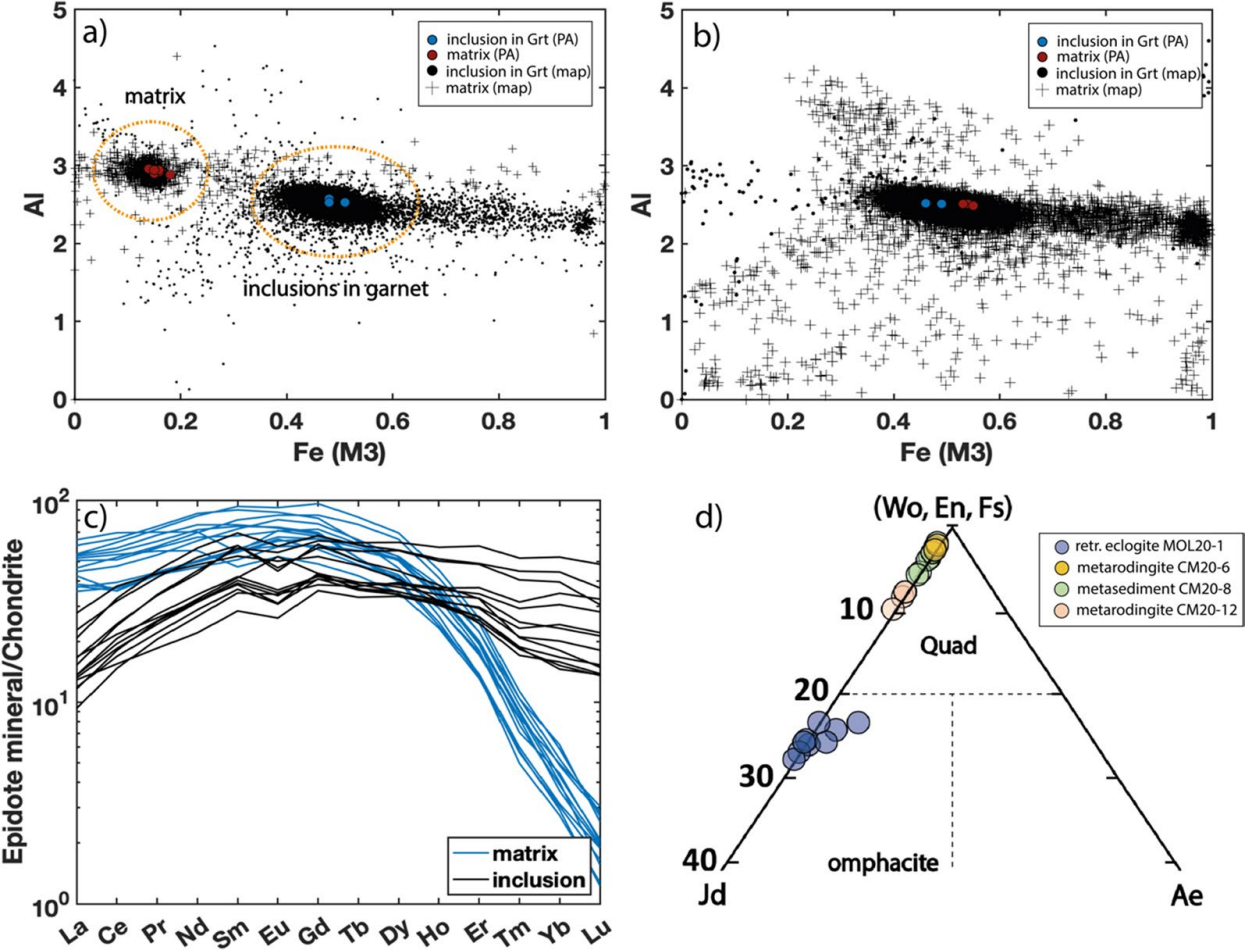
**a**, **b** Al vs Fe (M3) plot showing the compositional variation of the epidote minerals found in the matrix and as inclusions in garnet from retrogressed eclogite MOL20-1 (**a**) and MOL21-2 (**b**). Black symbols are data extracted from compositional maps in XMapTools and show all pixels classified as epidote. Coloured dots show EPMA analyses. **a** Matrix epidote has a higher X_Zo_ whereas inclusions have a higher X_Ep_ in sample MOL20-1. **b** Epidote included in garnet in sample MOL21-2 is zoned (see also Supplementary Fig. S3 & S4) whereas epidote in the matrix is unzoned. **c** Chondrite normalised REE (Boynton, 1984) pattern of epidote minerals occurring as inclusions in garnet and in the matrix of MOL20-1. **d** Composition of omphacite relicts from sample MOL20-1 and clinopyroxene compositions of sample CM20-6, CM20-8, and CM20-12. The compositions have been calculated from EPMA analyses

In the retrogressed eclogites MOL20-1 and MOL21-2 there is a difference in composition between epidote included in garnet and matrix grains (Fig. 10a and b and Supplementary File 1, Fig.S2 & S3). The epidote/clinozoisite inclusions in garnet core and mantle show varying compositions (extracted from the garnet map in XMapTools, Supplementary Fig. S3) from higher clinozoisite composition Ep_(0.39)_ to higher epidote composition Ep_(0.54)_. Some of the inclusions in sample MOL21-2 show a rim that is high in epidote composition ~ Ep_(0.85)_. The matrix clinozoisite, on the other hand, is not zoned and has an average composition of Ep_(0.16)_ (in MOL20-1) and Ep_(0.49)_ (in MOL21-2). Epidote/clinozoisite contained in garnet and in the matrix show a distinct REE composition (Fig. 10c). The inclusions show a relatively flat REE pattern and thus likely formed together or before the garnet core. The matrix grains, on the other hand, are enriched in LREE and MREE and are strongly depleted in HREE (La_N_/Lu_N_ = 0.29–2.00), suggesting formation when a significant amount of REE was already sequestrated in garnet, thus likely in equilibrium with the garnet rim.

#### Omphacite/Diopside

Relics of matrix omphacite in retrogressed eclogite sample MOL20-1 show minor chemical variations of Jd_(0.20)_ to Jd_(0.28)_ with an average composition of Di_(0.75)_, Jd_(0.25)_ and Ae_(0.01)_. Both metarodingites (CM20-6 and CM20-12) have a similar diopside average composition with X_Wo_ = 0.52, X_En_ = 0.42, and X_Fs_ = 0.07 (CM20-6) and X_Wo_ = 0.51, X_En_ = 0.44, and X_Fs_ = 0.05 (CM20-12). Clinopyroxene in metasediment CM20-8 has a composition of X_Wo_ = 0.52, X_En_ = 0.36, and X_Fs_ = 0.13. All pyroxene compositions are plotted in Fig. 10c and listed in the Supplementary Table S2.3 and S2.4.

#### Amphibole

**Fig. 11:**
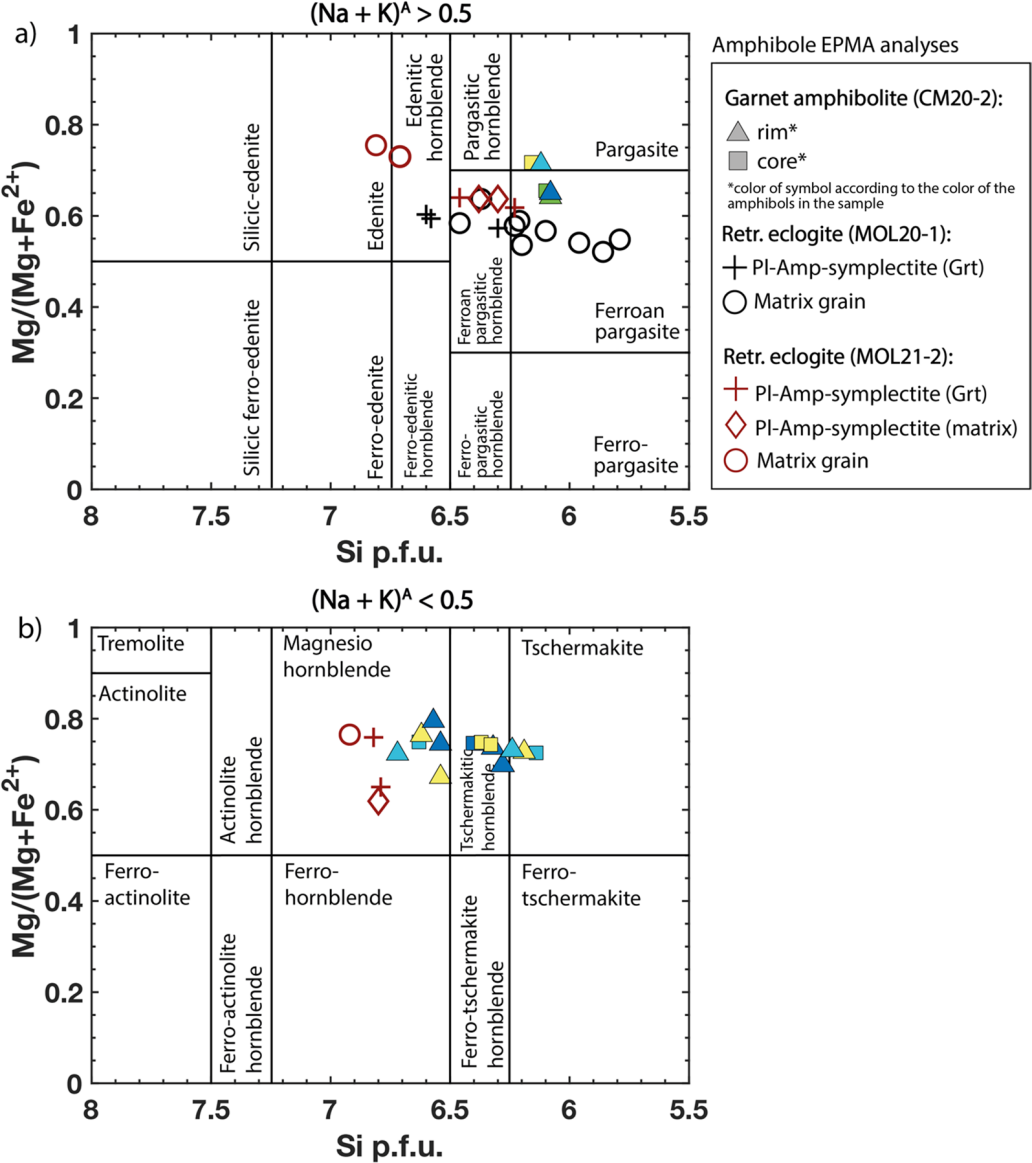
Calcic amphibole classification diagrams **a**, **b** after Leake et al., (1997)

All amphiboles are classified after Leake et al. (1997) and their compositions are shown in Fig. 11. Most amphiboles are hornblende with minor pargasite and edenite. Large amphibole grains around garnet and in the matrix of retrogressed eclogites show a composition of ferroan pargasite and ferroan pargasitic-hornblende in sample MOL20-1, and magnesio-, and edenitic-hornblende, edenite, and gedrite in sample MOL 21-2. The composition of amphibole in the amphibole-plagioclase symplectites around garnet is ferroan pargasite, magnesio-, ferroan pargasitic-, whereas in the matrix symplectite amphibole is edenitic hornblende and magnesio- and ferroan-hornblende.

Hypidiomorphic to xenomorphic amphibole grains of the garnet amphibolite CM20-2 show variation in their composition from (ferroan-) pargasite, (ferro-) tschermakite, and tschermakitic- to magnesio-hornblende unrelated to size, color or textural position.

#### Plagioclase

Plagioclase rimming large matrix clinozoisite grains in retrogressed eclogite MOL20-1 shows a high anorthite component of Ab_(0.07)_-An_(0.93)_. Plagioclase of the matrix Pl-Amp-symplectite shows a comparable higher albite-rich component of Ab_(0.62)_-An_(0.38)_ (MOL20-1) and Ab_(0.60)_-An_(0.40)_ (MOL21-2). In comparison, the Pl-Amp-symplectite around garnet has a higher anorthite component (Ab_(0.13)_-An_(0.84)_-San_(0.04)_). The composition of plagioclase of the spinel-anorthite symplectite around kyanite in sample MOL21-2 is Ab_(0.10)_-An_(0.90)_.

The matrix plagioclase of the garnet amphibolite (CM20-2) is rich in anorthite component (Ab_(0.01)_-An_(0.99)_). All plagioclase compositions are listed in the Supplementary Table S2.6

#### Polyphase inclusions

Polyphase inclusions in retrogressed clinozoisite-kyanite eclogite (MOL21-2) contain K-feldspar with An_(0.02)_-Ab_(0.00)-_San_(0.98)_ and albite-rich plagioclase with an An_(0.23)_-Ab_(0.76)_-San_(0.01)_. Plagioclase in polyphase inclusions in the retrogressed eclogite (MOL20-1) has a higher anorthite content of An_(0.35)_-Ab_(0.64)_-San_(0.01)_ compared to sample MOL 21-2. Both types of plagioclase inclusions have higher albite contents than matrix plagioclase. The hydrous Ca-Al silicate is rich in Al_2_O_3_ (18.34 wt.%), and CaO (8.70 wt.%) and has a K_2_O content of 1.93 wt.%. The average total oxide content is 86.74 wt.%, indicating a high water content. These chemical data were generated using the sampling tool in XMapTools and are consistent with EPMA spot measurements (Supplementary Table S2.6). This chemical composition suggests that this may be a Ca–zeolite (e.g. Ca-heulandite), which may replace an unknown precursor phase.

### U–Pb dating, trace element composition and Ti-thermometry of zircon

#### Retrogressed eclogites—MOL20-1 and MOL21-2

**Fig. 12:**
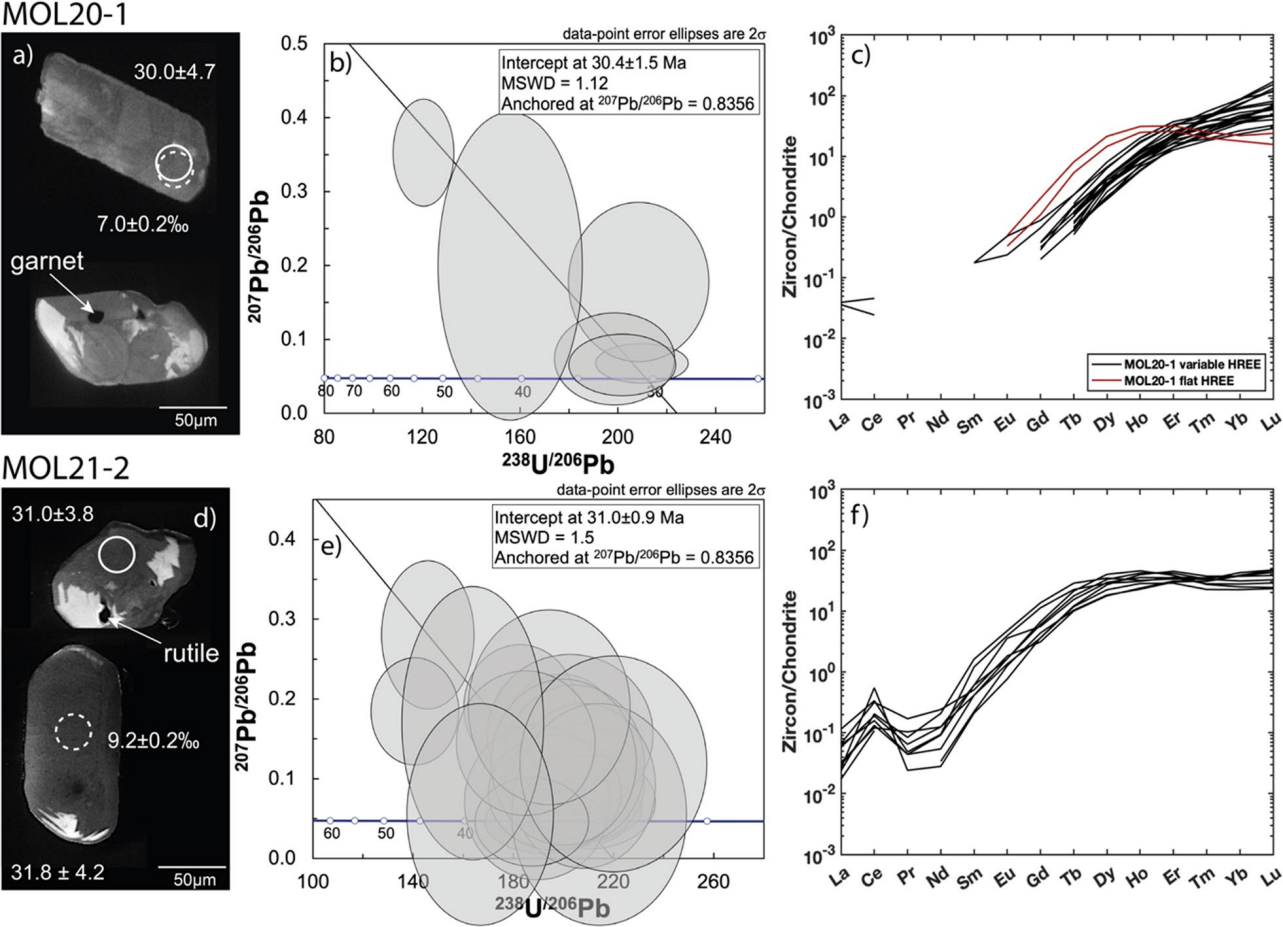
Zircon geochronology for retrogressed clinozoisite eclogites MOL20-1 and MOL21-2: **a**, **d** CC images of representative zircon crystals with circles indicating analysis spots (solid circle = U–Pb analyses with age ± Ma, dashed circle = δ^18^O analyses with values in ‰). The arrows indicate mineral inclusions. **b**, **e** TW diagram with intercept age (error ellipses are 2 sigma) anchored at ^207^Pb/^206^Pb = 0.8356. **c**, **f** Chondrite normalized (Boynton, 1984) REE content of zircon with Alpine age

Zircons extracted from samples MOL20-1 and MOL21-2 (Fig. 12a and b) are 50–200 µm in size, with elongated to rounded shape. In CC images these zircons show either a firtree or patchy zoning pattern or a dark center with a brighter rim. One inclusion each of garnet and rutile was found in zircon from MOL20-1, and rutile was found included in zircon from MOL21-2. In sample MOL20-1, the low U content (2–21 μg/g, Table S3) and the limited number of recovered zircons (N = 32), limited the number of center and rim analyses. Six zircon analyses from MOL20-1 yield a TW diagram intercept age of 30.4 ± 1.5 Ma (MSWD = 1.12, Fig. 12b) anchored to a modern common Pb. In sample MOL21-2, a large number of analyses define an age of 31.0 ± 0.9 Ma (MSWD = 1.5, Fig. 12e).

The REE compositions of the zircon in the two samples are different. In sample MOL20-1, most zircons show a REE pattern (Fig. 12c, Table S5) enriched in HREE relative to MREE (Gd_N_/Lu_N_ = 0.01–0.09) with only two analyses that are slightly flattened in HREE with respect to MREE. There is no correlation between different zircon textures and REE patterns. In sample MOL21-2, zircon REE patterns show a relatively flat MREE and HREE trend (Gd_N_/Lu_N_ = 0.07–0.29) with a pronounced positive Ce-anomaly (Fig. 12f). Zircon from this sample has higher MREE contents compared to zircon from sample MOL20-1. None of the analyses have a negative Eu-anomaly. The Th/U ratio for all zircon analyses of both samples are similarly low (< 0.05).

#### Garnet-amphibolite—CM20-2

**Fig. 13:**
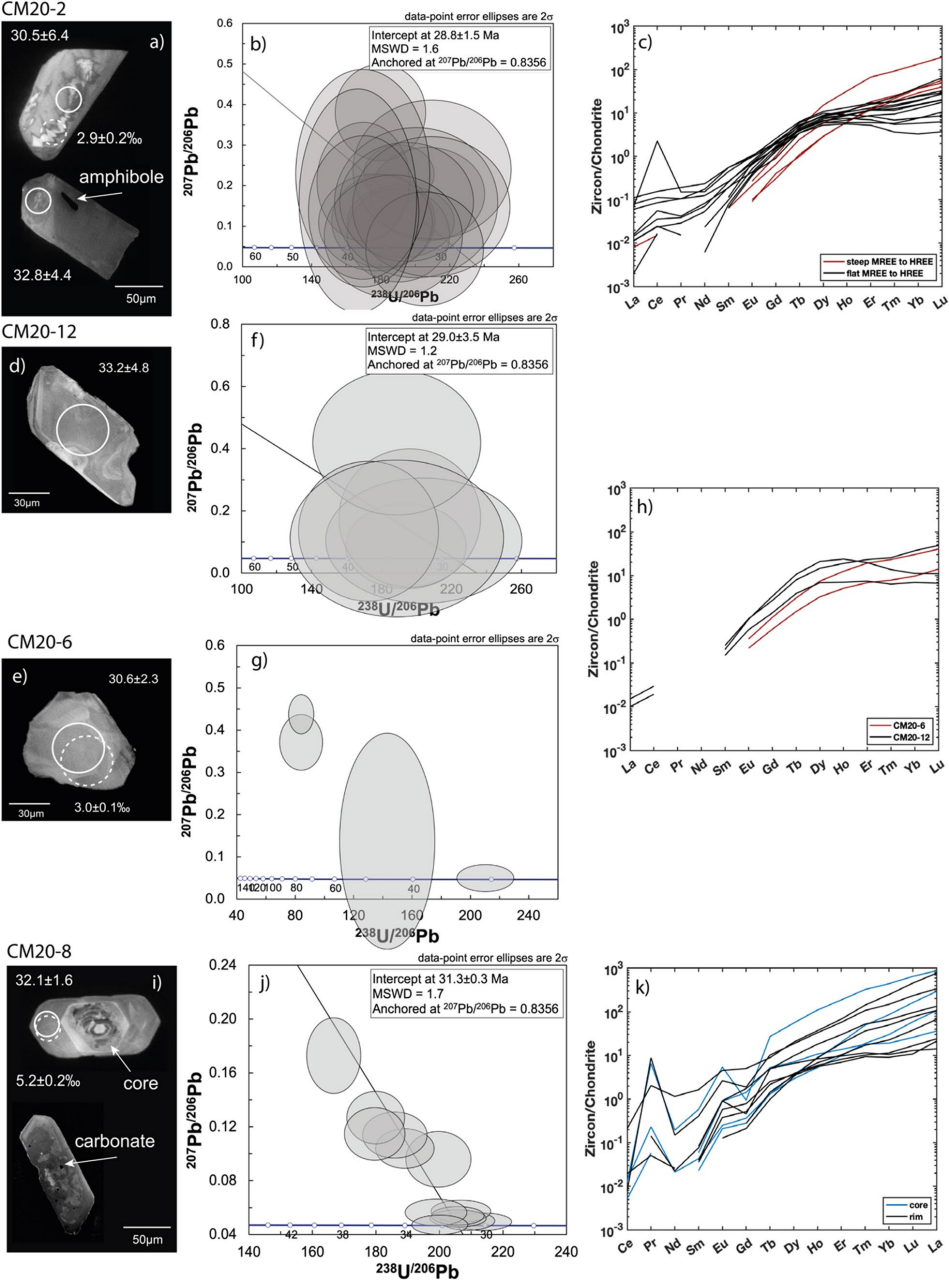
Zircon geochronology of different samples from Val Cama: garnet amphibolite CM20-2, metarodingites CM20-6 & CM20-12, and metasediment CM20-8: **a**, **d**, **e**, **i** CC images of representative zircon crystals with circles indicating analysis spots (solid circle = U–Pb analyses with age ± Ma, dashed circle = δ^18^O analyses with values in ‰). Arrows in **a** and **i** indicate mineral inclusions. **b**, **f**, **g**, **j** TW diagram with intercept ages anchored at ^207^Pb/^206^Pb = 0.8356 (error ellipses are 2 sigma). No statistically robust regression age could be calculated for sample CM20-6. **c**, **h**, **k** Chondrite normalized (Boynton, 1984) REE patterns of zircon with Alpine age and zircon cores with pre-Alpine ages

Zircon crystals from the garnet amphibolite CM 20-2 (Fig. 13a) are relatively small (50 to 150 µm in the main dimension) and vary from euhedral to anhedral in shape with predominantly patchy internal zoning and homogeneous rims. Other grains show a homogeneous center with a rim either with weak oscillatory zoning or CC-bright. The zircon grains contain inclusions of plagioclase and amphibole and have very low amounts of U (0.4–5.3 μg/g) and Th (b.d.l.—0.5 μg/g, Table S3). The zircon analyses (N = 17) yield an age of 28.8 ± 1.5 Ma with an MSWD of 1.6, defined by the intercept of the anchored regression in the TW diagram (Fig. 13b). The REE pattern (Fig. 13c) is characterized by a flat MREE to HREE pattern (Gd_N_/Lu_N_ = 0.03–0.25, Table S5) with a notable variation in HREE concentrations, with a pronounced to absent Ce-anomaly. Four analyses show a steep MREE to HREE pattern (Gd_N_/Lu_N_ ~ 0.01) with no Eu-anomaly (red lines Fig. 13c). There is no correlation between different zircon textures, REE patterns, and Th/U ratios.

#### Metarodingites—CM20-6 & CM20-12

The few zircons recovered from metarodingites are small (30–100 µm), anhedral, and elongated or rounded with patchy zoning (Fig. 13d and e). They have low to moderate U and Th contents of U = 2–92 μg/g and Th = b.d.l.–6.7 μg/g (the lowest values are found in CM20-6, Table S3 and S5). Six analyses of zircon from sample CM 20-12 define an imprecise anchored regression age of 29.0 ± 3.5 Ma with an MSDW of 1.2 (Fig. 13f). No age could be determined for the zircons from sample CM 20-6 as the analyses do not define a statistically significant regression on the TW diagram (Fig. 13g). The zircons of CM20-6 and one analyses of CM20-12 are enriched in HREE relative to MREE, and two zircon analyses of CM20-12 have relatively flat or even slightly depleted HREE (Fig. 13h, Table S5).

#### Metasediment—CM20-8

Zircon grains from the metasediment (Fig. 13i) are euhedral to subhedral and generally have a rounded core with inclusions of quartz, apatite, carbonate, and titanite. Cores are overgrown by CC-dark rims which are inclusion free. A few grains have a euhedral core with a weak oscillatory zoning. The zircon cores have significantly higher U (24–927 μg/g) and Th (1–172 μg/g) contents with ^206^Pb/^238^U dates ranging from 138 to 1736 Ma (Table S3). The youngest concordant age is 265 ± 6.9 Ma.

Zircon rims are relatively poor in U (37–139 μg/g) and Th (0.2–1.8 μg/g) and yield an age of 30.9 ± 0.4 Ma (MSWD = 1.3) with an initial ^207^Pb/^206^Pb of 0.673 ± 0.024 (2σ) defined by free regression on the TW diagram. This initial Pb value differs from the model common Pb of 0.8356 at 0 Ma (Stacey & Kramers, 1975). The regression anchored at ^207^Pb/^206^Pb = 0.8356 gives an age of 31.3 ± 0.3 Ma (MSWD = 1.7, Fig. 13j). Both ages overlap within uncertainty. Because of consistency with other analyses and poor dispersion along the regression, the anchored regression lower intercept is taken as the best age estimated.

The REE analyses of zircons from the metasediment show no systematic difference between core and rim (Table S5). They have highly variable patterns ranging from flattened or concave MREE and flat to enriched HREE, with a negative to no Eu-anomaly (Eu/Eu* = 0.08–0.81) and pronounced to weak positive Ce-anomaly (Fig. 13k).

The Ti-in-zircon temperature was calculated for all zircon analyses using the pressure-dependent calibration of Crisp et al. (2023), which contained a pressure dependence (Table S5). For the Moleno retrogressed eclogites the calculation was made assuming pressure of 3.0 and 2.3 GPa, which are the two extreme values for the range of pressure estimated for the HP assemblage in these samples. The estimated temperatures are higher for sample MOL21-2 (~ 780 and 740 °C although with a relatively large SD of 65 °C) than for sample MOL20-1 (~ 700 and 670 with a SD of 45 °C).

The Ti-in-zircon temperatures for the garnet amphibolite CM20-2 were calculated at 1 GPa and have an average of 610 ± 23 °C (1SD) with one outlier.

### Zircon oxygen isotopes

**Fig. 14:**
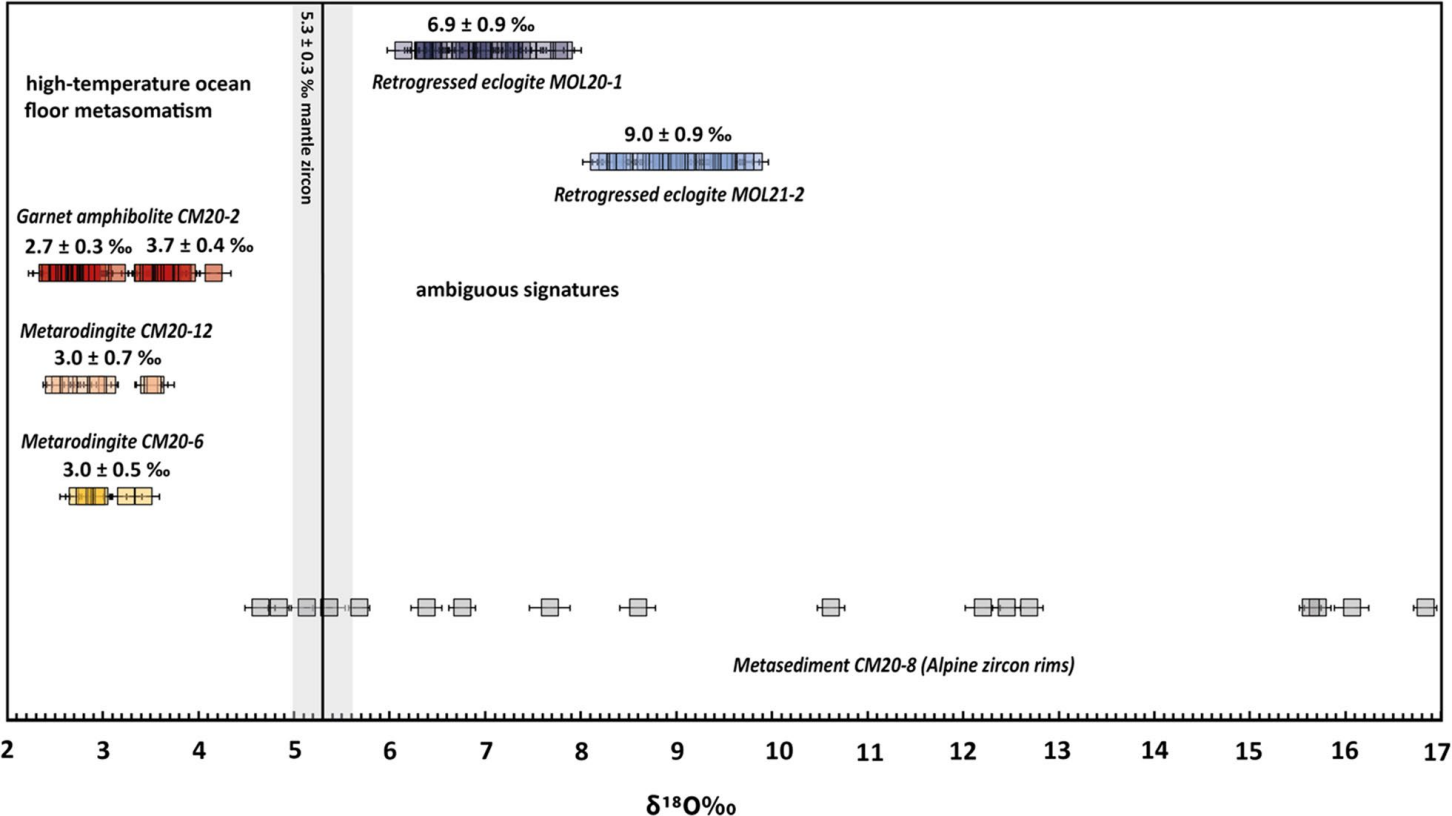
Oxygen isotopic composition of Alpine zircons from metamafic rocks expressed as δ^18^O values relative to VSMOW. Error bars are 2 sigma. The light grey bar shows the δ^18^O value of unaltered mantle zircons (Valley, 2003). The low δ^18^O values for metarodingites and garnet amphibolite indicate seafloor metasomatism of the protolith. The elevated δ^18^O values of retrogressed eclogites could be due to different processes (see text for discussion)

The zircon oxygen isotopic composition was determined for retrogressed eclogites, garnet amphibolite, metarodingites and a metasediment (Fig. 14 and l Supplementary Table S6). The zircon δ^18^O values from the retrogressed clinozoisite eclogite (MOL20-1, N = 28) range from 6.1 to 7.8 ‰ with an average of 6.9 ± 0.9 ‰ (2SD, two outliers excluded). The δ^18^O analyses for the retrogressed clinozoisite-kyanite eclogite (MOL21-2, N = 35) show higher values compared to MOL20-1, ranging from 8.2 to 9.7 ‰ (average 9.0 ± 0.9 ‰).

Zircons from the Val Cama metamafic rocks have significantly lower δ^18^O values than the retrogressed eclogites. Zircon δ^18^O values from the garnet amphibolite (CM20-2) range from 2.4 to 4.2 ‰: zircon cores show systematically higher values with an average of 3.7 ± 0.4 ‰ (2SD), while the rims have an average of 2.7 ± 0.3 ‰ (2SD). One core analysis falls into the group with the lower average and for two analyses the textural domain is unclear. The average δ^18^O for zircon from both Cama metarodingites is comparable: 3.0 ± 0.5 ‰ (CM20-6) and 3.0 ± 0.7 ‰ (CM20-12, one outlier at 4.0 ‰.). Zircon δ^18^O values from the calcsilicate rock CM20-8 show a large spread from 4.5 to 16.8 ‰, with a comparable range for core and rim analyses. Within the same grain, core and rim analyses generally have different values.

### Rutile U–Pb dating and thermometry

**Fig. 15:**
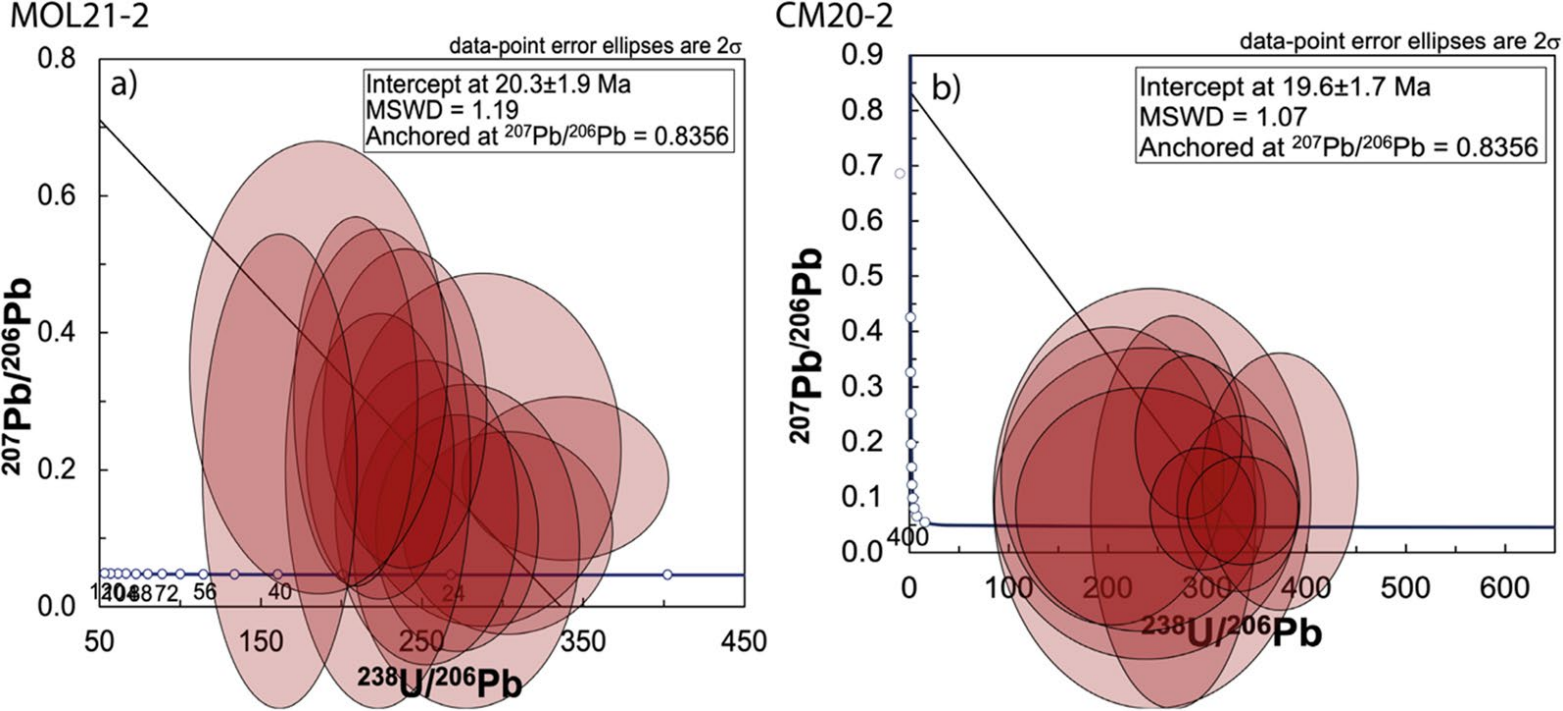
TW diagrams of rutile U–Pb analyses anchored to present-day common Pb. Error ellipses are 2 sigma. **a** Retrogressed clinozoisite-kyanite eclogite MOL21-2. **b** garnet amphibolite CM20-2

Rutile grains in the retrogressed eclogite MOL21-2 show no zoning in BSE images and have low U contents (0.5–3.7 μg/g). The intercept age anchored for initial Pb defines an age of 20.3 ± 1.9 Ma with an MSWD of 1.19 (Fig. 15a). Rutile grains in the garnet amphibolite CM20-2 also have low U contents (0.31–4.69 μg/g). The ten analyses that define best an intercept age of 19.6 ± 1.7 Ma anchored to a modern initial ^207^Pb/^206^Pb of 0.8356 (Stacey & Kramers, 1975) with a MSWD of 1.07 (Fig. 15b).

The U content of rutile from the Val Cama metarodingites is particularly low (0.1–0.6 μg/g for CM20-6 and 0.2–5.2 μg/g for CM20-12) and thus the analyses contain a particularly high proportion of common Pb, which prevents obtaining a meaningful regression age in the TW plot for both samples (Supplementary File 1, Fig. S4).

The Zr content of rutile from the retrogressed eclogite MOL21-2 and garnet amphibolite CM20-2 range from 415 to 205 µg/g and from 424 to 230 µg/g, respectively. The average of the five highest concentrations are 380 and 333 µg/g, respectively. Both samples contain metamorphic zircon and quartz and thus the Zr-in-rutile thermometry can be applied. The measured Zr mass fraction corresponds to 730 °C at 2.3 GPa and 770 °C at 3.0 GPa for MOL21-2 and 660 °C at 1.0 GPa and 680 °C at 1.4 GPa for CM20-2 using the calibration of Tomkins et al. (2007). A temperature calculation for the metarodingites was not performed, because no quartz was observed in these samples.

## Discussion

### Mafic–ultramafic rocks from the oceanic crust

Serpentinization of the oceanic lithosphere is a concurrent process with the rodingitisation of mafic rocks of the oceanic crust (Coleman et al., 1967; Laborda-López et al., 2018). The coexistence of ultramafic rocks and metarodingites is an index of oceanic alteration and is commonly observed in the more complete metaophiolites in the Western Alps e.g., in Zermatt (Li et al., 2004), Erro Tobbio (Hermann et al., 2000; Scambelluri & Rampone, 1999), Aosta Valley (Panseri et al., 2008) and in the Central Alps e.g., Cima di Gagnone (Evans et al., 1979, 1981; Pfiffner & Trommsdorff, 1998) and Val Malenco (Müntener & Hermann, 1996). Therefore, the field relationships of metarodingites associated with chlorite-harzburgites, the dehydration product of antigorite-serpentinites, in Val Cama and Valle di Moleno indicates that these rocks have undergone seafloor metasomatism and thus represent remnants of the oceanic crust.

The mafic–ultramafic suite in Val Cama is associated with calcsilicate rocks and carbonate lenses interpreted as the sedimentary cover of the former mafic/ultramafic oceanic crust. The age of detrital zircon grains in the calcsilicate rocks provides information on the age of sedimentation. The zircon cores in sample CM20-8 have discordant ^206^Pb/^238^U dates ranging from 138 to 1736 Ma, which do not define a single population. These observations are consistent with detrital zircons of variable age that have undergone partial resetting of the U–Pb system. The maximum deposition age of the sediment is given by the youngest concordant age (Gehrels, 2014), which is 265 ± 7 Ma (Supplementary Table S3.6). The high variability in REE, oxygen isotopes and ages suggest that these zircon cores may have different origins and be derived from erosion of different basement rocks. The limited dataset requires caution in the interpretation, but a post-Permian depositional age is consistent with the formation of this sequence in the Jurassic Alpine Tethys.

Similar rock associations of oceanic sediments and mafic–ultramafic rocks in the Central Alps exist at Cima di Gagnone (Pfiffner & Trommsdorff, 1998). Unfortunately, it was not possible to obtain any information on the age of the protolith of the mafic rocks, and therefore the correlation with fragments of the Piemont-Ligurian Ocean is based solely on lithological arguments.

Further evidence for oceanic alteration of the protolith of the mafic rocks is provided by the oxygen isotope compositions of zircons that yield Alpine ages of the garnet-amphibolite and metarodingites (Val Cama). The average δ^18^O of 2.7–3.0 ‰ is below the typical mantle zircon value of 5.3 ‰ (Valley, 2003). Such low δ^18^O values have been reported for bulk rock analyses of altered gabbros of the Omani, Corsican and Zermatt-Saas ophiolites (Cartwright & Barnicoat, 1999; Gregory & Taylor, 1981; Miller et al., 2001) and for bulk rock and zircon δ^18^O analyses of eclogitic metagabbros in the Alps (Barnicoat & Cartwright, 1997; Bocchio et al., 2004; Rubatto & Angiboust, 2015). These values are interpreted as high-temperature seafloor metasomatism of the mafic protolith (Miller et al., 2001). The possibility of lowering the δ^18^O values by infiltration of fluids derived from the adjacent altered oceanic lithosphere during prograde subduction (Angiboust et al., 2014) is less likely because it would imply a large amount of fluid at high pressure. Therefore, we suggest that the metamafic rocks of Val Cama acquired a low δ^18^O from high-temperature seafloor metasomatism and the Alpine zircon inherited the bulk δ^18^O signature during metamorphic (re)crystallization. In contrast, the eclogite lenses at Trescolmen, further north in the Adula nappe, have a protolith age that is older than Mesozoic (561 ± 22 Ma, Liati et al., 2009; Sandmann et al., 2014). The Trescolmen rocks have bulk oxygen isotope values that are not shifted from the mantle value (5.5 ± 0.6 ‰ Eiler, 2001) and are not associated with ultramafic rocks or rodingites (Wiesli et al., 2001), thus representing mafic rocks of the basement, unlike the relicts of the Piemont-Ligurian Ocean in Val Cama.

The significantly elevated δ^18^O values of 6.9 ± 0.9 ‰ and 9.0 ± 0.9 ‰ in zircons of the Moleno retrogressed eclogites are ambiguous. Low-temperature metasomatism at the seafloor could have shifted the δ^18^O values of mafic rocks to higher values (Gregory & Taylor, 1981; Miller et al., 2001). Additional possible evidence for alteration of the protolith of retrogressed eclogites by ocean floor metasomatism could be provided by the abundance in the mineral assemblage of Ca- and Al-rich minerals such as epidote minerals, kyanite, Ca-rich plagioclase, and a high X_Grs_ in garnet cores. The enrichment of Ca and Al and depletion of Na and Si is typical of mafic rocks undergoing epidote alteration (Evans et al., 1979; Laborda-López et al., 2018; Li et al., 2004). Alternatively, the elevated δ^18^O values in retrogressed eclogites could have been caused by fluids released during prograde subduction of adjacent ultramafic rocks, if altered at low temperatures, as well as metasediments, which typically have δ^18^O values > 8 ‰ (Hoefs, 2009). The absence of K-phases in the mafic rocks argue against the infiltration of a sediment-derived fluid. Modification of bulk rock and isotopic compositions of the mafic lenses related to fluid release from serpentine breakdown in the ultramafic rocks remains a possibility that requires further investigation.

### Peak metamorphic conditions

#### Valle di Moleno

The inferred peak mineral assemblages of Grt + Czo + Omp + Qtz + Rt + Zr (MOL20-1) and Grt + Ky + Czo + Rt + Zr + Qtz (MOL21-2) are consistent with HP metamorphism. Unfortunately, there are no omphacite relics preserved in sample MOL21-2 and no kyanite in sample MOL20-1that would allow pressure estimates. Nevertheless, the peak assemblage provides clear evidence for equilibration at eclogite facies conditions. There is no evidence for coesite or pseudomorphs after coesite in garnet inclusions, suggesting that the rocks equilibrated below the coesite stability field. Zoisite and its polymorph clinozoisite can be stable under high pressure and high temperature up to eclogite-facies conditions in a Ca-rich mafic rock (Enami et al., 2004). The peak mineral assemblages are also consistent with the experimental stability of mineral assemblages of zoisite eclogites (Poli & Schmidt, 1998), whose stability field lies between ~ 2.3 and 3.0 GPa and ~ 600–750 °C (Poli, 2016; Poli & Schmidt, 1998 and references therein). Furthermore, the peak mineral assemblages of the investigated samples are comparable to assemblages of zoisite eclogites, zoisite-bearing eclogites or eclogitic veins containing zoisite that have been identified in HP to UHP terrains such as: the Sanddal area, North-East Greenland, which exposes zoisite eclogites with a peak assemblage of Grt + Omp + Ky + Zo + Rt ± Phe ± Qtz and modelled peak conditions of 2.4 ± 0.1 GPa at 830 ± 30 °C (Cao et al., 2020); the Dabie-Shan terrane, China, which has eclogites with a peak assemblage of Grt + Omp + Czo + Rt and estimated peak conditions of 2.4 GPa at 700 ± 20 °C (e.g. Castelli et al., 1998); the Sulu UHP eclogites, China, have the peak assemblage of Grt + Omp + Zo + Ky + Phe + Rt ± Coe/Qtz with estimated peak conditions of > 2.8 GPa at ~ 800 °C (Yao et al., 2000).

The retrogressed eclogites coexist with chlorite-peridotite containing chlorite pseudomorphs after garnet, suggesting that the associated ultramafic rocks may have reached the stability field of garnet during subduction, requiring a minimum pressure of 2.0 GPa (Lakey & Hermann, 2022). Pseudomorphic replacement of garnet by chlorite has been observed in chlorite-peridotite of the nearby locality of Alpe Arami (e.g. Pfiffner & Trommsdorff, 1998) as well as at Monte Duria (Tumiati et al., 2018) and is likely related to the Barrovian metamorphic overprint. We suggest based on peak pressure mineral assemblages, the stability field of kyanite-zoisite eclogites, and the mafic–ultramafic rock association that the retrogressed eclogites reached pressure conditions between 2.3 and 3.0 GPa.

Peak temperatures were calculated using the Grt-Cpx thermometer (Supplementary Table S2.13; calibration after Ellis & Green, 1979) using garnet rim and omphacite relics in the matrix assuming equilibrium. The composition of the garnet rim was determined using garnet maps and the sampling tool in XMapTools. The values obtained from the map are in agreement with the results of the microprobe point analyses. The calculated temperatures range from 700 ± 50 °C at 2.3 GPa and 730 ± 50 °C at 3.0 GPa at the inner rim to 850 ± 50 °C at 2.3 GPa and 870 ± 50 °C at 3.0 GPa at the outer rim. The increasing temperature from the garnet mantle to the garnet rim is correlated with the increasing X_Prp_ towards the garnet rim. The calculated prograde to peak temperatures for the retrogressed eclogite sample MOL21-2 with the Zr-in-rutile thermometer are 730 °C at 2.3 GPa and 770 °C at 3.0 GPa and overlap with the temperatures of the Grt-Cpx thermometer. Similarly, the Ti-in-zircon temperatures for the Moleno samples are mainly between 650 and 850 °C. The transition of chlorite + orthopyroxene to garnet + olivine in the associated peridotite occurs at 780–800 °C at 2.0 to 3.0 GPa according to the experiments of Lakey and Hermann (2022). In summary, the peak assemblages of retrogressed eclogites, petrographic observations, and the application of two geothermometers suggest that the mafic rocks of Valle di Moleno underwent HP-HT metamorphism with peak pressures that lie between 2.3 and 3.0 GPa and temperatures most likely in the range 780–850 °C.

The estimated pressure conditions in this study are consistent with pressure conditions determined for mafic rocks in the Cima-Lunga unit and the southern Adula nappe, including Cima di Gagnone, Alpe Arami, Alpe Caurit and Monte Duria, which range between 2.4 and 3.0 GPa (Brouwer et al., 2005; Dale & Holland, 2003; Ernst, 1981; Tumiati et al., 2018), and for a metasediment from Cima di Gagnone (~ 2.7 GPa, Piccoli et al., 2022). Peak pressures for ultramafic rocks in these localities (except Alpe Caurit) range from 2.8 to 3.2 GPa (Nimis & Trommsdorff, 2001; Pellegrino et al., 2020) and tend to be slightly higher compared to mafic rocks. The estimated peak temperature range of 700–850 °C is comparable in its higher values to the temperature estimate for mafic–ultramafic rocks for the above localities, suggesting that these rock types were heated to temperatures of ~ 800 °C while at HP conditions.

#### Val Cama

The garnet amphibolite CM20-2 shows a typical high-pressure amphibolite-facies assemblage of hornblende, minor plagioclase, rutile and strongly retrogressed garnet porphyroblasts. Therefore, the temperatures calculated with the Zr-in-rutile thermometer are interpreted as the peak temperature of amphibolite-facies metamorphism. The zircon in sample CM20-2 is also interpreted to form at amphibolite facies (see below) and the Ti-in-zircon temperature for this sample is 610 ± 23 °C assuming 1.0 GPa, which is in agreement with the absence of a high-pressure, high-temperature eclogite stage preceding the amphibolite facies metamorphism. The calcsilicate metasediment as well as the rodingites display an assemblage that is not sensitive to variations in temperature and pressure. The ultramafic rocks consist of olivine + orthopyroxene + chlorite + magnetite ± talc indicating pressures of ~ 1.4 GPa and temperatures of ~ 650 °C (Viera-Duarte et al., 2023).

### Origin of polyphase inclusions

The polyphase inclusions hosted in the garnet mantle of retrogressed eclogites of the Valle di Moleno contain quartz, K-feldspar, Na-rich plagioclase and a hydrous Ca-Al silicate. These minerals display an irregular intergrowth and the inclusions vary in size from ~ 15 µm to ~ 200 µm, some of which have a negative crystal shape (Fig. 3c). The granitic mineralogy and texture of mineral phases of polyphase inclusions in the Moleno retrogressed eclogites, is consistent with the nanogranites described by Cesare et al. (2009) and other studies of UHP eclogites e.g. in the Dabbie-Sulu orogen (Gao et al., 2012) or Saidenbach eclogites (Borghini et al., 2023). Interestingly, the nanogranites of these studies as well as polyphase inclusions in our samples show a restricted number of mineral phases and no rock-forming phases (also discussed by Borghini et al., 2023), which is interpreted as crystallization of trapped melts.

### Partial melting during HP metamorphism

The MSI (multiphase solid inclusions) interpreted as melt inclusions are found at the boundary between the mantle and rim of garnet, which shows bell-shaped zonation of major elements (Fig. 8f) typical of prograde to peak growth (Hollister, 1966). The decrease in HREE from garnet core to rim (Fig. 8) is further consistent with Rayleigh fractionation during prograde growth (Rubatto et al., 2020; Spandler et al., 2003). Taken together, these observations suggest that partial melting occurred under HP conditions. The garnet rim adjacent to garnet mantle containing the melt inclusion has a formation temperature of ~ 700 ± 50 °C at 2.3 GPa and ~ 730 ± 50 °C at 3.0 GPa which increases to ~ 800 ± 50 °C towards the rim. The polyphase inclusions contain K-feldspar, whereas no K-phase was observed in the matrix of the retrogressed eclogites suggesting that phengite might have been involved in the partial melting.

Partial melting of phengite-zoisite eclogites due to the breakdown of hydrous phases such as phengite, paragonite or zoisite has been described in the Dabie-Sulu UHP belt (Chen et al., 2014; Gao et al., 2012; Liu et al., 2013). Several melting reactions have been proposed in experimental studies: Phe + Cpx + Qtz = Grt + Kfs + melt (Patiño Douce, 2005) or Phe + Cpx + Qtz + H_2_O = Grt + Ky + melt (Hermann & Green, 2001). The melting reaction with the breakdown of phengite and clinozoisite: Phe + Czo + Cpx + H_2_O = Grt + Ky + melt could also be considered. Partial melting of metamafic rocks at 2.3–3.0 GPa, 780–850 °C is facilitated by infiltration of an aqueous fluid to lower the solidus temperature (Carter et al., 2014; Hermann et al., 2006). Such an aqueous fluid could be provided by the associated ultramafic rocks of Valle di Moleno. Chlorite pseudomorphs after garnet in the chlorite-harzburgites provide evidence that the ultramafic rocks may have undergone the chlorite-out reaction during subduction (Chl + Opx = Ol + Grt + H_2_O; Lakey & Hermann, 2022; Padrón-Navarta et al., 2013).

### Timing of metamorphic events

#### High-pressure melting

Zircons from the clinozoisite-kyanite retrogressed eclogite (MOL21-2) yield an age of 31.0 ± 0.9 Ma and are characterized by a relatively flat HREE pattern with no Eu-anomaly. This pattern is typical for zircon formed under eclogite-facies conditions with coexisting garnet and in the absence of plagioclase (Rubatto, 2002, 2017). Inclusions of rutile further support formation in a HP assemblage. Therefore, we interpret the age of 31.0 ± 0.9 Ma to date eclogite-facies metamorphism.

The retrogressed clinozoisite eclogite (MOL20-1) contains some zircons with relatively flat HREE patterns and no Eu-anomaly. However, the remaining REE analyses show variable HREE enrichment and no Eu-anomaly, which may indicate zircon growth during prograde garnet growth or retrograde garnet resorption. The domains with the two distinct REE patterns cannot be resolved by age but suggest that zircon grew over variable P–T conditions around the pressure peak. Furthermore, these zircons show inclusions of HP phases such as garnet and rutile. We propose that the age of this sample (30.4 ± 1.5 Ma) also dates eclogite-facies metamorphism, but rather a late stage of the HP evolution. This is in line with the slightly lower Ti-in-zircon temperatures (average 700 and 670 at 3.0, and 2.3 GPa, calibration of Crisp et al., 2023) estimated for this sample compared to MOL21-2 (average 780 and 740 at 3.0, and 2.3 GPa, Table S5).

The presence of melt inclusions and the disturbed garnet zoning in the retrogressed eclogites (Fig. 8) suggests partial melting by fluid infiltration during HP metamorphism. Given that zircon is generally reactive to melt (dissolution and crystallization, e.g. Rubatto 2017), zircon from sample MOL21-2 may have grown from a melt and its age of ~ 31 Ma is tentatively interpreted as the date of HP melting.

Our study documents younger HP ages in Valle di Moleno compared with U–Pb zircon data from previous studies (35–36 Ma) in the Cima-Lunga unit and Adula nappe (Gebauer et al., 1996; Nimis & Trommsdorff, 2001). On the other hand, zircon rims of eclogites from the central Adula nappe at Trescolmen (1.8–2.4 GPa, 600–650 °C, Meyre et al., 1997, 1999), which show flat REE patterns with no Eu-anomaly, also have an age of ~ 32 Ma (Liati et al., 2009) comparable to what was found at Moleno.

Lutetium-Hf dating of garnet in mafic rocks from the central Adula nappe gives ages of 38.8 ± 4.3 Ma and 37.1 ± 2.8 Ma (Herwartz et al., 2011; Sandmann et al., 2014). The authors suggest that these are maximum ages of Alpine HP metamorphism because they found mixed Variscan and Alpine garnet populations that may not be fully equilibrated to Alpine HP conditions. The Lu–Hf age for garnet at Alpe Arami is 34.1 ± 2.8 Ma (Sandmann et al., 2014), within uncertainty of the zircon ages from the Moleno retrogressed eclogites. The variable trace element composition of zircon in MOL20-1 suggests that zircon did not necessarily grow under peak pressure conditions and that the age rather dates a late stage of HP metamorphism.

#### Amphibolite-facies metamorphism

Zircons in sample (CM20-2) contain inclusions of typical amphibolite-facies minerals such as plagioclase and amphibole. The zircon age of 28.8 ± 1.5 Ma is therefore interpreted to date amphibolite-facies metamorphism. The REE pattern of zircon (Fig. 13c) is similar to the eclogite-facies zircon of Moleno (Fig. 12c) probably because both samples contain a high amount of garnet. In the Moleno retrogressed eclogites, the absence of a negative Eu-anomaly is attributed to the absence of plagioclase at eclogite-facies conditions. The Cama garnet-amphibolite has very low amounts of plagioclase (3%) and therefore the absence of an Eu anomaly in zircon cannot be used as an indication of eclogite-facies conditions, but the amphibolite-facies mineral inclusions in zircon are considered more diagnostic. The high variability of HREE may indicate that zircon grew during partial resorption of the garnet. The age of the metarodingite CM20-6 of 29.0 ± 3.5 Ma overlaps within uncertainty with the age obtained from the garnet amphibolite and is also interpreted to date amphibolite-facies metamorphism. The timing of amphibolite-facies metamorphism in this study (~ 29 Ma) is consistent or slightly younger than previous zircon U–Pb ages (30–33 Ma) for the northern part of the Southern Steep Belt and in the Cima-Lunga unit and the Adula nappe (Gebauer et al., 1996; Hermann et al., 2006; Rubatto et al., 2009, Tagliaferri et al., 2023).

The age determined for the zircon rims of the Cama metasediment (31.3 ± 0.3 Ma) overlaps with the HP ages of the Moleno retrogressed eclogites as well as the amphibolite-facies age of the garnet amphibolite. The zircon REE pattern and inclusions do not provide sufficient evidence for a link to specific P–T conditions. Thus, the age of the metasediment cannot be assigned to a specific metamorphic stage.

Rutile in samples MOL21-2 and CM20-2 yields U–Pb ages that overlap at ~ 20 Ma and are significantly younger than the ages of HP and amphibolite-facies metamorphism. Rutile crystallized at elevated temperatures as documented by the Zr-in-rutile temperatures ranging between 650 and 700 °C (at 1.4 GPa) for the garnet amphibolite (CM20-2) and between 700 and 750 °C (2.3 GPa) and 720–780 °C (3.0 GPa) for the retrogressed eclogite (MOL21-2). It has been shown that rutile subjected to metamorphic temperatures above the closure temperature for Pb diffusion (< 550–600 °C, Cherniak, 2000; Kooijman et al., 2010) can retain crystallization temperature information, which is decoupled from the U–Pb age information (Ewing et al., 2013, 2015; Kooijman et al., 2012; Luvizotto et al., 2009). As the Zr-in-rutile temperatures of the retrogressed eclogite and garnet amphibolite are all above the closure temperature for Pb in rutile, they rather document rutile crystallization during prograde to peak metamorphic conditions. We therefore interpret the rutile U–Pb ages as dating the cooling (< 550–600 °C) after Barrovian amphibolite-facies metamorphism.

#### Exhumation rates

**Fig. 16:**
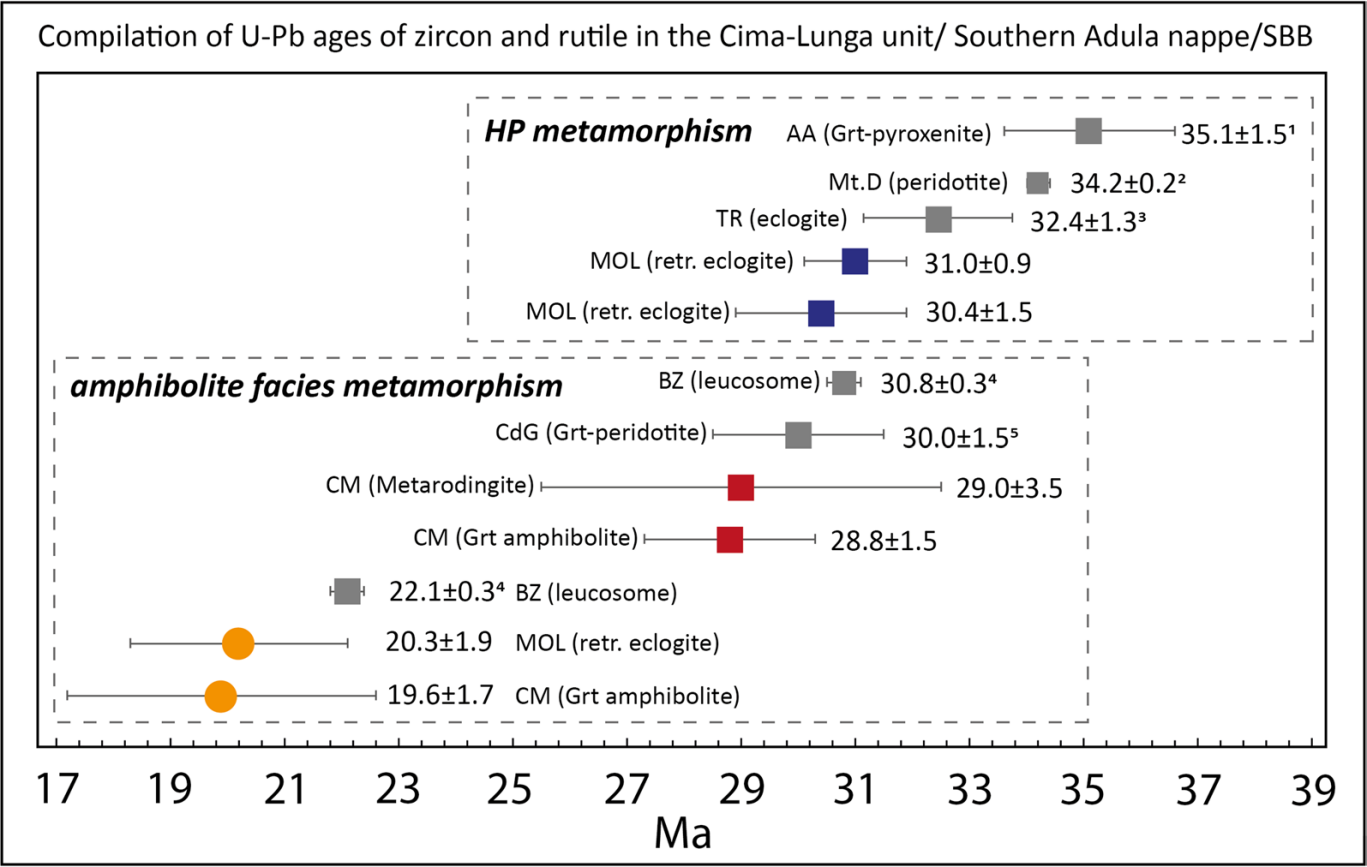
**a** Compilation of U–Pb ages of zircon and rutile from rocks of the Cima-Lunga unit and Southern Adula nappe. Ages are sorted by high-pressure and amphibolite-facies metamorphism. Coloured boxes are ages from this study with uncertainty (2 sigma) and grey boxes are ages from the literature. AA: Alpe Arami; Mt.D: Mt. Duria; TR: Trescolmen MOL = Moleno; CM: Cama; CdG: Cima di Gagnone; BZ: Bellinzona. References are indicated by numbers: 1: Gebauer (1996), 2: Hermann et al. (2006), 3: Liati et al. (2009), 4: Rubatto et al. (2009), 5: Gebauer (1994)

**Fig. 17:**
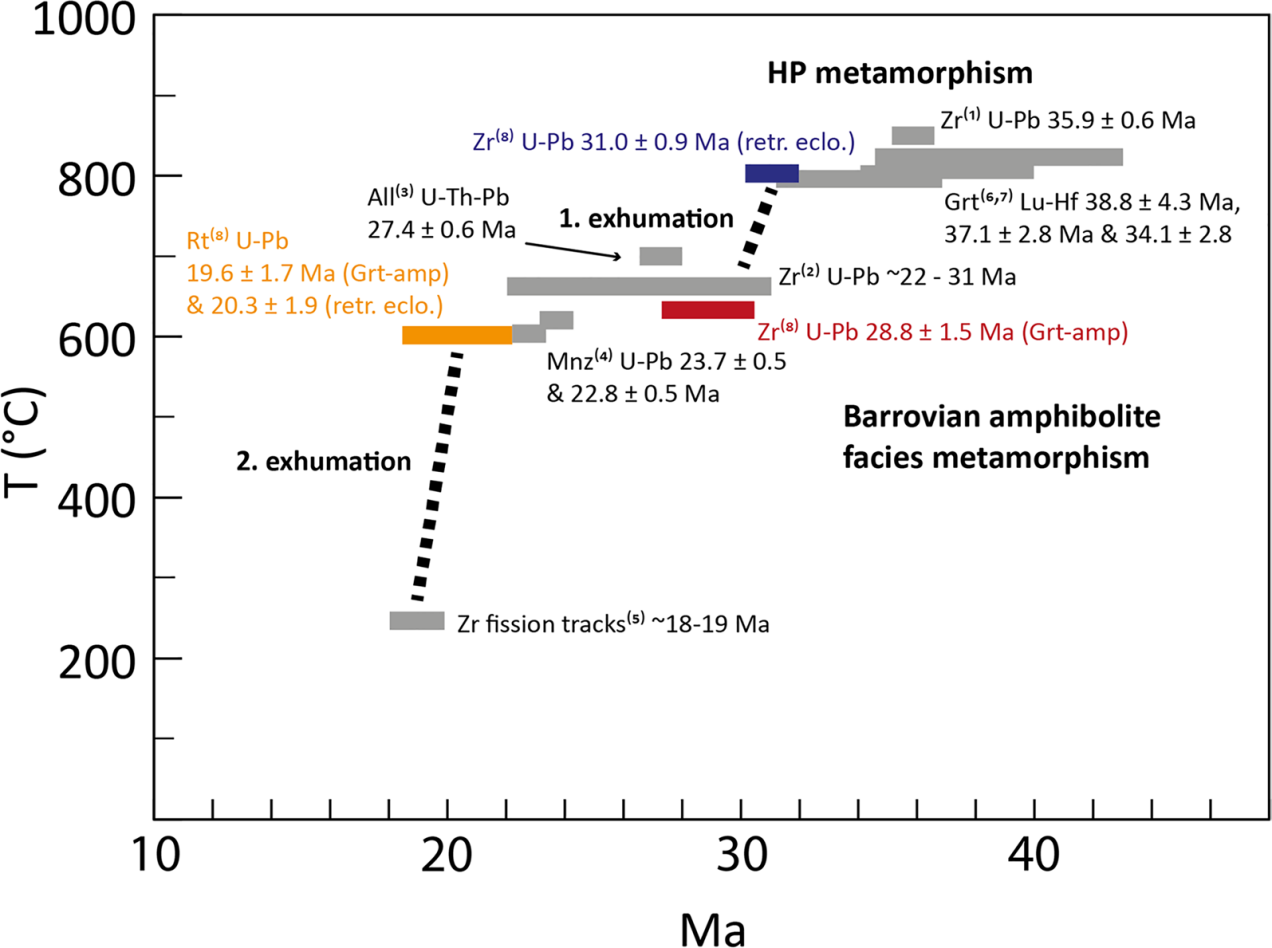
Cooling path in the Cima-Lunga unit and Southern Adula nappe showing two exhumation stages (modified from Rubatto et al., 2009). The ages used in this diagram are from (1): Corvò et al. (2021), (2): Rubatto et al. (2009), (3): Boston et al. (2017), (4): Köppel and Grünenfelder (1975), (5): Hurford (1986), (6): Herwartz et al. (2011), (7): Sandmann et al. (2014), (8): this study

The ages of the HP (~ 31 Ma) and amphibolite-facies metamorphism (~ 29 Ma) show a close temporal transition between these two stages (Figs. 16 and 17). This implies that a rapid decompression and cooling from HP conditions of 2.5–3.0 GPa and 800 °C (e.g. Brouwer et al., 2005; Nimis & Trommsdorff, 2001; Piccoli et al., 2022) to amphibolite-facies conditions of 1.0–1.4 GPa and 650 °C (Burri, 2005.; Dale & Holland, 2003; Piccoli et al., 2022) occurred within 1–2 Ma with corresponding exhumation rates of 3–6 cm/yr.

Furthermore, the younger rutile cooling ages suggest that the mafic rocks of Valle di Moleno and Val Cama resided at amphibolite-facies conditions for ~ 10 Ma. This time span has been reported by Rubatto et al. (2009) as the period during which migmatisation occurred in the Southern Steep Belt. Other constraints on the Barrovian amphibolite-facies metamorphism in these tectonic units come from monazite ages (23–28 Ma, Köppel & Grünenfelder, 1975) and allanite ages (23–30 Ma, Boston et al., 2017; Gregory et al., 2012). This age coincidence suggests that the mafic lenses were exhumed from HP conditions into the migmatites and their evolution was subsequently coupled to that of the migmatites.

A possible geodynamic scenario that accounts for the rapid exhumation is slab break-off. During the onset of the continent–continent collision, differences in buoyancy between the downgoing oceanic lithosphere and the continental lithosphere entering the subduction channel caused stresses and deformation within the downgoing slab, which resulted in the break-off at a depth of ~ 100 km (Davies & von Blanckenburg, 1995; Schlunegger & Kissling, 2015). The Moleno mafic–ultramafic suite would have been situated at a lesser depth at the subducted, former European continental margin. The association of peridotite-eclogite and felsic rocks or sediments might have resulted in a negative buoyancy needed for the fast exhumation. Indeed, high-pressure paragneisses with P–T conditions similar to Moleno have been recently reported from the nearby Cima di Gagnone (Piccoli et al., 2022). Hot asthenospheric material could enter the gap between the oceanic lithosphere and the detached slab, leading to an increase in thermal gradient in the oceanic lithosphere, which can explain the observed heating in the retrogressed eclogites of ~ 800 °C in the late stage of HP metamorphism. A similar heating stage has also been observed in subducted paragneisses at Cima di Gagnone (Piccoli et al., 2022). An alternative model to explain a fast transition from subduction-related to Barrovian metamorphism is slab extraction (Froitzheim et al., 2003). This model has been developed to account for structures and metamorphic gradients observed in the Adula nappe (Fig. 1) and accounting for the subduction of two ocean basins, the Piemont-Ligurian Ocean and the Valais trough. The delamination of parts of the lower crust and lithospheric mantle of the subducted microcontinent (Briançonnais) separating the two ocean basins would have resulted in the fast rebound of the former European continental margin (see Fig. 4 in Froitzheim et al., 2003) at the transition of subduction to continental collision.

A second phase of rapid exhumation must have occurred at ~ 20 Ma, as indicated by the U–Pb cooling ages of rutile at Moleno (20.3 ± 1.9 Ma) and Cama (19.6 ± 1.7). Early Miocene ages are also recorded in rutile from the western part of the Lepontine Dome (21.5 ± 2.5 Ma, Boston et al., 2017). The age of the youngest leucosome in the Southern Steep Belt is 22.12 ± 0.25 Ma (Valle d’Arbedo, Rubatto et al., 2009), indicating that temperatures were still above the wet granite solidus (650 °C) at 22 Ma. At ~ 20 Ma, the temperature must have been < 550–600 °C, the maximum closure temperature for Pb in rutile. The oldest zircon fission track ages from Ponte Brolla are 18.9 ± 0.9 Ma (Hurford, 1986), indicating cooling below 250 °C. These ages suggest that there was a first stage of cooling with a cooling rate of about 100 °C in 2 Ma followed by a faster cooling of 300 °C in the next 2 Ma (see also Boston et al., 2017 for a data compilation supporting fast cooling across the entire Central Alps). Considering an average geothermal gradient of 30 °C/km this would translate into an exhumation rate of 3 cm/y. Such rapid cooling and exhumation must have been accommodated by tectonic exhumation of the Central Alps and is probably related to mylonitisation and backthrusting along the Insubric Line (Schmid et al., 1987).

## Conclusion

The mafic–ultramafic suites in Valle di Moleno and Val Cama, within the Southern Steep Belt of the Central Alps, are derived from oceanic crust that has been hydrothermally altered. The mafic–ultramafic lens of Val Cama is associated with metasedimentary rocks that are probably of Mesozoic origin. During Alpine subduction, the clinozoisite-(kyanite) retrogressed eclogites of Valle di Moleno underwent fluid-assisted melting at HP conditions, as suggested by polyphase inclusions consisting of plagioclase, K-feldspar and quartz in garnet. Dating of zircon showing a typical eclogite REE pattern yields an age of 31.0 ± 0.9 Ma. There is no evidence for eclogite-facies metamorphism in the Val Cama mafic–ultramafic lens. Zircon crystals from a garnet amphibolite contain inclusions of plagioclase and amphibole. U–Pb dating of these zircons gives an age of 28.8 ± 1.5 Ma, indicating a rapid transition from subduction-related metamorphism to Barrovian metamorphism in about 2 Ma. This requires fast exhumation tectonics, possibly related to slab break-off or slab extraction. U–Pb cooling ages of rutile in Valle di Moleno (20.3 ± 1.9 Ma) and Val Cama (19.6 ± 1.7 Ma) are within error and only slightly younger than the youngest leucosome in the Sothern Steep Belt. This suggests a second episode of very rapid cooling and exhumation that is probably related to vertical movements along the Insubric line.

### Supplementary Information

Supplementary Material 1.

Supplementary Material 2.

Supplementary Material 3.

Supplementary Material 4.

Supplementary Material 5.

Supplementary Material 6.

Supplementary Material 7.

## Data Availability

All data generated or analysed during this study are included in this published article and its supplementary information tables and files.
