# Supplementary material for: A rapid transition from subduction to Barrovian metamorphism: geochronology of mafic–ultramafic relicts of oceanic crust in the Central Alps, Switzerland
Source: Swiss J Geosci. 2024 Aug 30;117(1):15. doi: 10.1186/s00015-024-00462-7 (PMC11364590; doi:10.1186/s00015-024-00462-7)
Supplement: Supplementary file 7 — Supplementary Material 7. [file 15_2024_462_MOESM7_ESM.docx]

**Supplementary File 1**


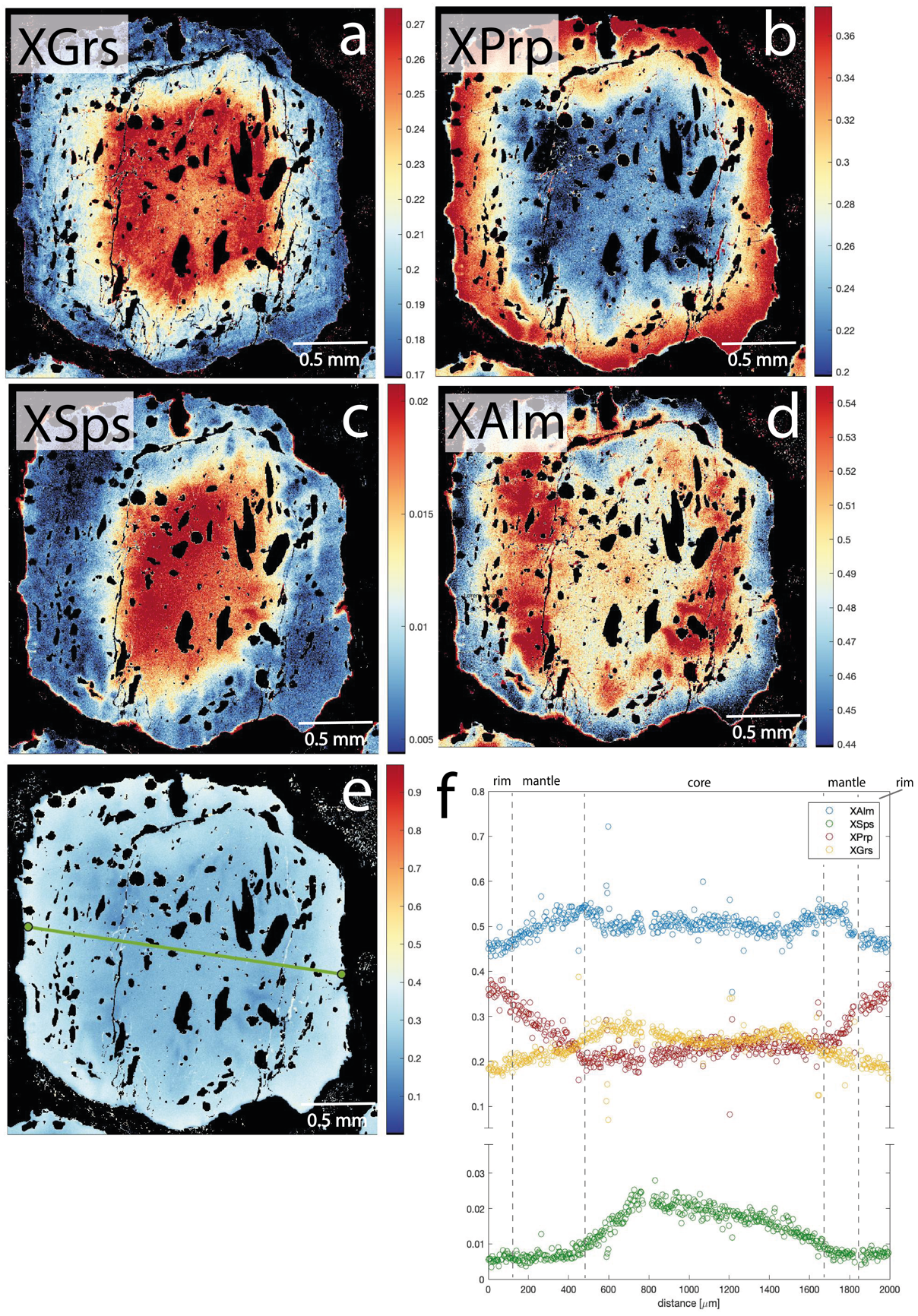


Fig. S1 a)-d) garnet endmember maps of retrogressed kyanite-clinozoisite eclogite MOL21-2. e) BSE image of the same garnet. f) Chemical zoning of garnet along a rim-core-rim profile for the same garnet as in a)-e) and extracted from the compositional maps.


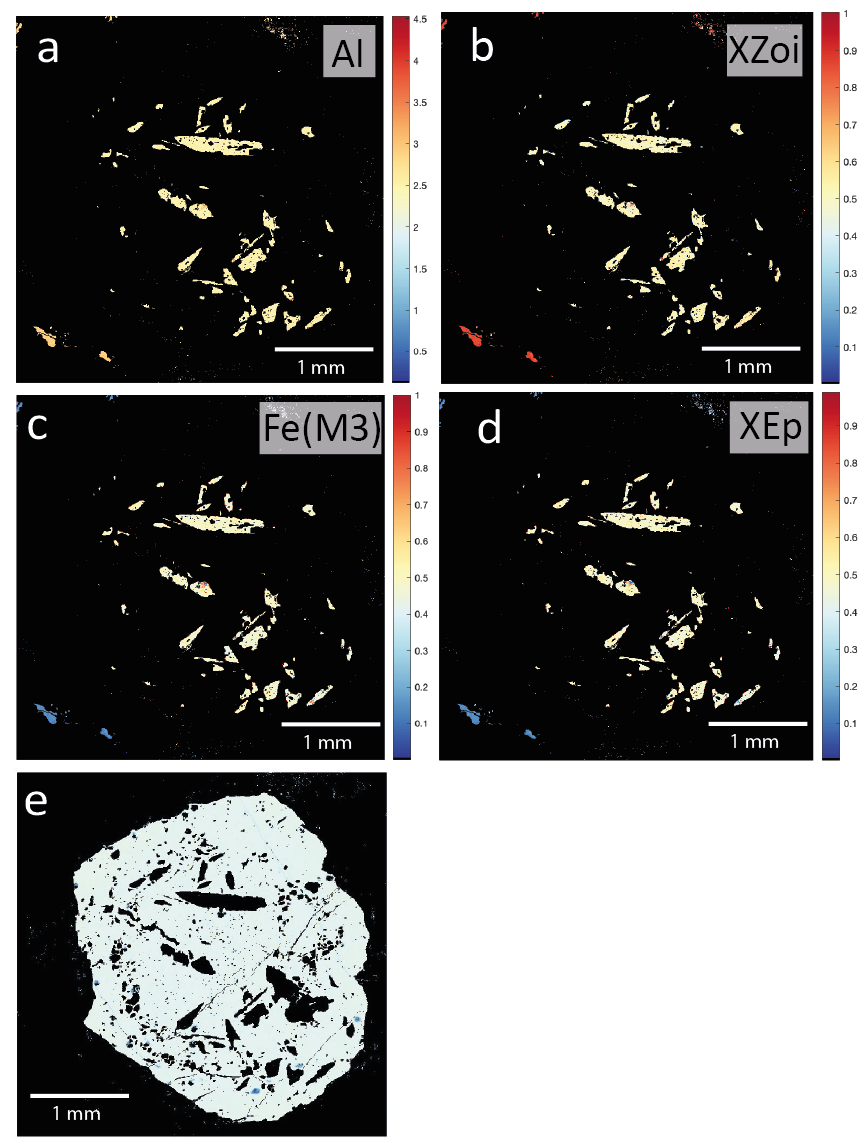


Fig. S2 Zoisite/epidote composition map (a&c) and endmember maps (b&d) of retrogressed clinozoisite eclogite MOL20-1. e) show the same map as in a)-d) with visible garnet grain.


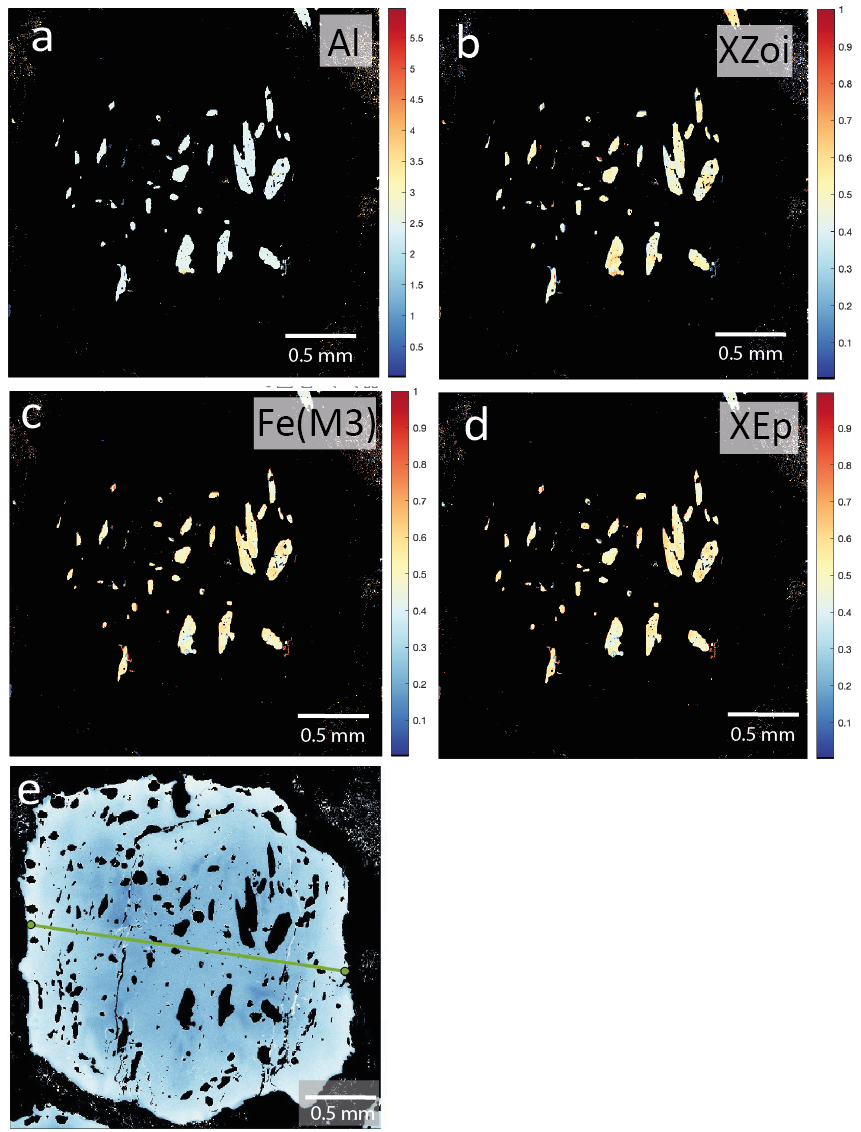


Fig. S3 Zoisite/epidote composition map (a&c) and endmember maps (b&d) of retrogressed clinozoisite-kyanite eclogite MOL21-2. e) show the same map as in a)-d) with garnet visible.


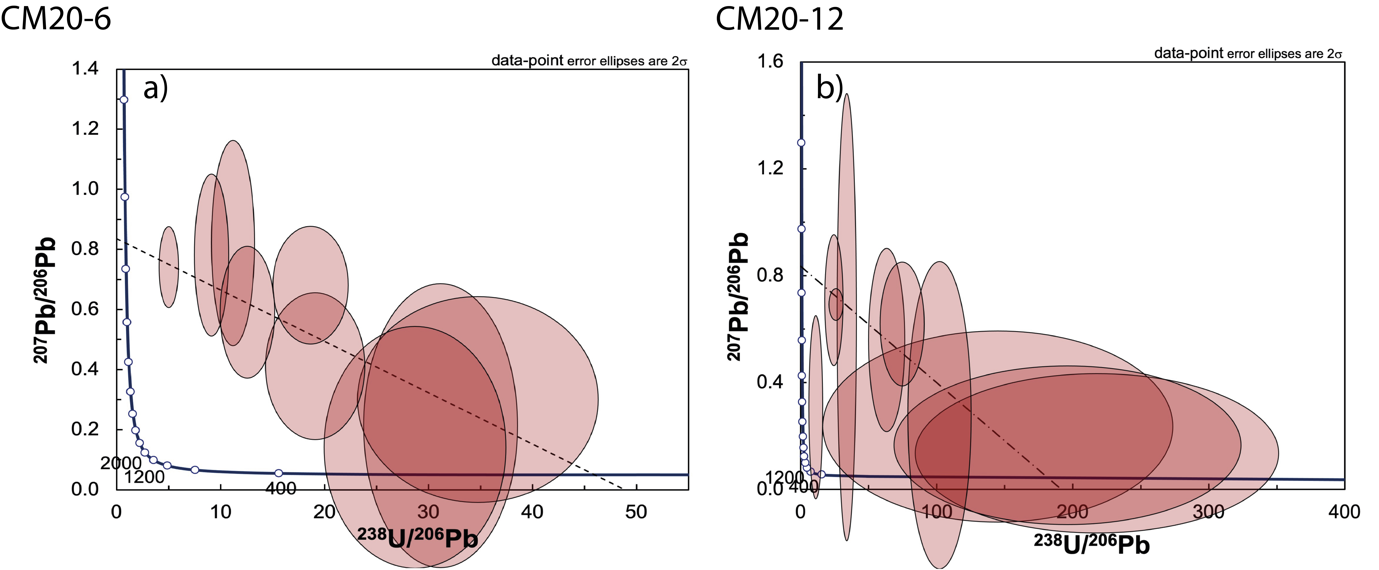


Fig. S4 TW diagrams of rutile U-Pb analyses anchored to present-day common Pb. Error ellipses are 2 sigma. a)&b) Metarodingites CM20-6 and CM20-12. No age could be determined for these samples due to too large uncertainties.
